# Immune regulation of intestinal-stem-cell function in *Drosophila*

**DOI:** 10.1016/j.stemcr.2022.02.009

**Published:** 2022-03-17

**Authors:** Minjeong Shin, Meghan Ferguson, Reegan J. Willms, Lena O. Jones, Kristina Petkau, Edan Foley

**Affiliations:** 1 Department of Medical Microbiology and Immunology Faculty of Medicine and Dentistry University of Alberta Edmonton, Edmonton, AB Canada; 2 Department of Cell Biology Faculty of Medicine and Dentistry University of Alberta Edmonton, Edmonton AB, Canada

**Keywords:** Drosophila, intestinal stem cell, immunity, proliferation, differentiation, IMD

## Abstract

Intestinal progenitor cells integrate signals from their niche, and the gut lumen, to divide and differentiate at a rate that maintains an epithelial barrier to microbial invasion of the host interior. Despite the importance of evolutionarily conserved innate immune defenses to maintain stable host-microbe relationships, we know little about contributions of stem-cell immunity to gut homeostasis. We used *Drosophila* to determine the consequences of intestinal-stem-cell immune activity for epithelial homeostasis. We showed that loss of stem-cell immunity greatly impacted growth and renewal in the adult gut. In particular, we found that inhibition of stem-cell immunity impeded progenitor-cell growth and differentiation, leading to a gradual loss of stem-cell numbers with age and an impaired differentiation of mature enteroendocrine cells. Our results highlight the importance of immune signaling in stem cells for epithelial function in the adult gut.

## Highlights

• The immune deficiency (IMD) pathway is active in *Drosophila* intestinal progenitors
• Inhibition of IMD in progenitors impairs progenitor-cell proliferation
• Blocking IMD in progenitors impairs generation of mature epithelial cells

## Teaser

In this article, Foley and colleagues show that activation of IMD in intestinal progenitor cells regulates stem-cell proliferation and differentiation in the adult *Drosophila* midgut. Blocking progenitor-cell IMD impairs epithelial renewal and impacts the generation of mature enteroendocrine cells.

## Introduction

The intestine is an important contact point between animals and their environments. Intestinal epithelial cells regulate nutrient acquisition, microbiota tolerance, immune education, and pathogen elimination, and disruptions to epithelial homeostasis are linked to inflammatory diseases and cancers. As the epithelium contains a heterogenous population of specialist cell types, it is essential that we understand the mechanisms by which individual lineages regulate intestinal cell proliferation, differentiation, and renewal.

Intestinal epithelial cell (IEC) lineages vary by animal, but data from *Drosophila*, zebrafish, mice, and humans indicate evolutionary conservation of cell-type composition (Brugman, 2016; Buchon et al., 2013a; Lickwar et al., 2017; Miguel-Aliaga et al., 2018; Nguyen et al., 2015; Wallace et al., 2005). Typically, the epithelium is maintained by proliferative, multipotent intestinal stem cells (ISCs) that self-renew and generate all mature epithelial cell types. Most differentiated cells are columnar enterocytes, a cell type specializing in capture and digestion of lumenal nutrients. Secretory-cell-type complexity varies from animal to animal. In flies, the secretory lineage consists solely of hormone-producing enteroendocrine cells. Fish and mammals have mucus-secreting goblet cells in addition to the enteroendocrine population, and mammals also have long-lived, antimicrobial-peptide-producing Paneth cells that neighbor ISCs in basal crypts. ISCs integrate cues from their niche to divide and differentiate at a rate that replenishes dying epithelial cells. Notch, epidermal growth factor (EGF), Wnt, and bone morphogenetic protein (BMP) signal transduction pathways are important regulators of ISC proliferation and differentiation in vertebrates and invertebrates (Spit et al., 2018). Recent studies uncovered roles for immune signaling in ISC survival, growth, and differentiation. For example, vertebrate ISCs express major histocompatibility complex (MHC) class II molecules, and presentation of self-peptides by ISCs appears to be a critical aspect of intestinal invasion and epithelial destruction in graft-versus-host disease (Biton et al., 2018; Fu et al., 2019; Takashima et al., 2019). Likewise, ISCs are enriched for expression of the germline-encoded peptidoglycan receptor NOD2 (Nigro et al., 2014). NOD2 protects ISCs against reactive oxygen species toxicity (Levy et al., 2020), and mutations in NOD2 are associated with Crohn’s disease and intestinal tumorigenesis (Couturier-Maillard et al., 2013). Despite established requirements for immune-signaling pathways in the maintenance of intestinal health, it is unclear if innate defenses act specifically in progenitors to regulate epithelial homeostasis. We consider this an important knowledge gap given the central role of intestinal progenitors in building and maintaining the entire epithelium.

*Drosophila melanogaster* are widely used to characterize intestinal immunity and homeostasis. The adult fly intestine is a pseudostratified epithelium that is maintained by multipotent ISCs (Micchelli and Perrimon, 2006; Ohlstein and Spradling, 2006). The majority of ISC divisions are asymmetric, producing a new ISC, and are transient cell types that generate terminally differentiated epithelial cells. In most cases, ISC divisions generate a post-mitotic enteroblast that differentiates as an enterocyte in response to Notch signals (Bardin et al., 2010; Guo and Ohlstein, 2015; Ohlstein and Spradling, 2007). Collectively, ISCs and enteroblasts are classified as the intestinal progenitor compartment in flies. In the absence of cues from Notch, ISCs transition through a pre-enteroendocrine state to generate mature enteroendocrine cells that can be sub-classified into functional groups based on intestinal localization and hormone expression patterns (Biteau and Jasper, 2014; Guo and Ohlstein, 2015; Zeng and Hou, 2015). In the fly gut, bacterial diaminopimelic acid-type peptidoglycan (PGN) activates immune responses via the immune deficiency (IMD) pathway, a germline-encoded defense with similarities to vertebrate tumor necrosis factor receptor signaling (Buchon et al., 2009b, 2013a; Myllymäki et al., 2014). Detection of extracellular PGN by the PGN recognition protein LC (PGRP-LC) receptor, or intracellular PGN by the related PGRP-LE receptor, converges on a signaling complex that includes the Imd protein, the adaptor protein Fas-associated death domain (FADD), and the Caspase-8 homolog, Dredd. Dredd removes thirty N-terminal amino acids from Imd, initiating molecular events that activate c-Jun N-terminal kinase, and the p100/105 nuclear factor κB (NF-κB) ortholog Relish (Rel) (Leulier et al., 2000; Stoven et al., 2003; Stöven et al., 2000). Thus, Dredd-mediated processing of Imd is essential for IMD pathway activation, and expression of a non-cleavable Imd variant (ImdD30A) blocks host responses to PGN in cell culture and *in vivo* (Kim et al., 2014; Paquette et al., 2010). In the fly intestine, Rel and c-Jun N-terminal kinase initiate transcriptional responses that include regionalized expression of antimicrobial peptides, regulators of metabolism, and genes associated with growth and differentiation (Broderick et al., 2014; Buchon et al., 2009b, 2009a; Dutta et al., 2015; Hung et al., 2020). Earlier work uncovered significant differences between the responses of mature epithelial cell types to IMD activation. For example, infection-dependent activation of IMD in enterocytes results in antimicrobial peptide expression and extrusion of damaged cells (Buchon et al., 2009b; Dutta et al., 2015; Zhai et al., 2018). In contrast, activation of IMD in enteroendocrine cells by bacterial lactate modifies lipid metabolism in neighboring enterocytes (Kamareddine et al., 2018). Notably, genomic studies demonstrated expression of IMD pathway components in intestinal progenitor cells (Dutta et al., 2015; Hung et al., 2020). However, the contributions of progenitor-specific IMD activity to intestinal homeostasis are unexplored.

We took advantage of the genetic accessibility of flies to ask if progenitor-specific IMD affects intestinal homeostasis in *Drosophila*. Specifically, we used genomic and physiological assays to determine the consequences of blocking IMD in intestinal progenitors. We found that inhibition of progenitor-cell IMD had significant effects on ISC proliferation, progenitor compartment composition, and generation of mature enteroendocrine cells. As germline-encoded immune responses are known modifiers of vertebrate intestinal epithelial growth, we believe our findings are of general relevance to understanding how host immune responses control stem-cell function in the intestine.

## Results

### IMD regulates the intestinal progenitor-cell transcriptome

**Figure 1:**
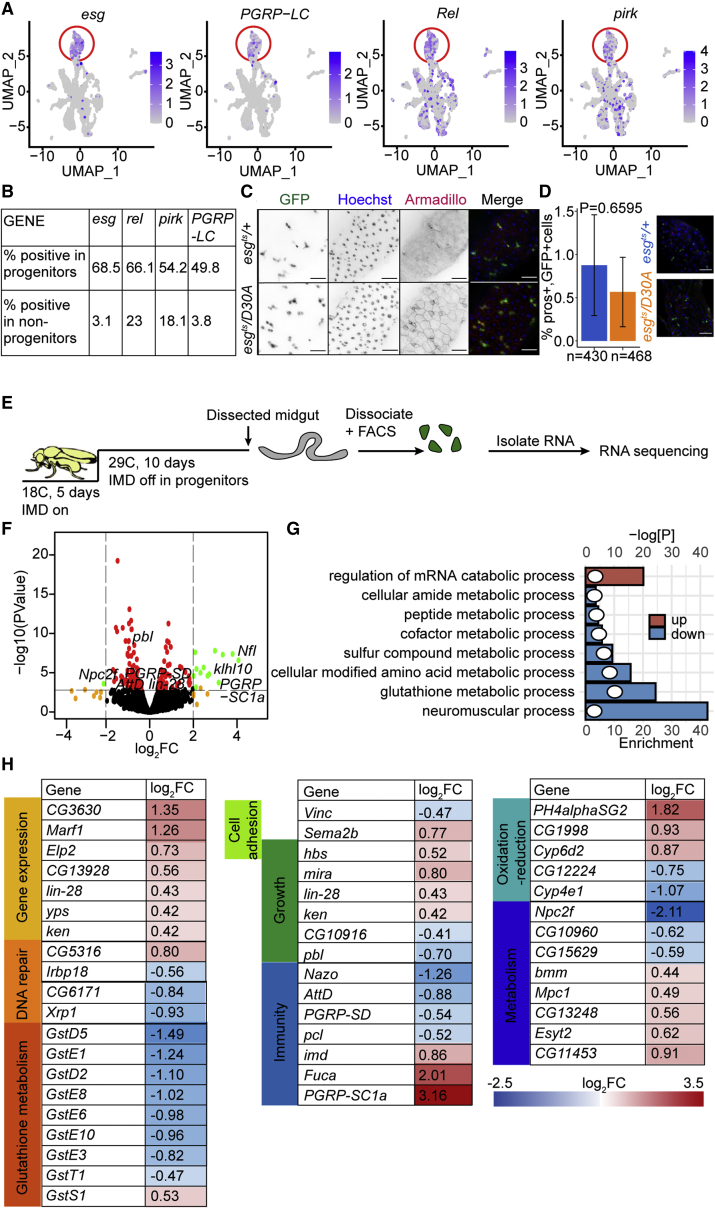
IMD regulates the intestinal progenitor-cell transcriptome (A) Uniform manifold approximation and projection (UMAP) plot of intestinal epithelial cells isolated from 10-day-old *esg*^*ts*^ flies showing expression of the progenitor marker *esg* and the IMD pathway components *PGRP-LC*, *Rel*, and *pirk*. Progenitors are circled in red. (B) Quantification of the percentage of progenitors and non-progenitors that express the indicated genes. (C) Visualization of GFP, DNA (Hoechst), and the beta-catenin ortholog in intestines of 10-day-old *esg*^*ts*^/*+* and *esg*^*ts*^/*UAS-imdD30A (esg*^*ts*^/*D30A*) flies. Scale bars represent 25 μm. (D) Quantification of the percentage of GFP-positive cells that express the enteroendocrine cell marker *prospero* in *esg*^*ts*^/*+* (n = 20) and *esg*^*ts*^/*D30A* (n = 22) flies. *esg*^*ts*^/*+* (n = 430) and *esg*^*ts*^/*D30A* (n = 468) n = number of GFP+ cells. Representative images are shown with DNA in blue, *esg*+ cells in green, and *prospero*-positive enteroendocrine cells in red. Significance measured using Student’s t test. Scale bars represent 25 μm. (E) Schematic representation of an experimental strategy to quantify gene expression in progenitor cells purified from *esg*^*ts*^/*+* and *esg*^*ts*^/*D30A* flies. Each experiment was performed in triplicate. (F) Volcano plot showing relative changes (x axis) and significance (y axis) in gene expression of purified progenitors from *esg*^*ts*^/*D30A* flies compared with age-matched *esg*^*ts*^/*+* controls. Genes are color-coded to indicate significance and relative gene expression changes. (G) Gene Ontology analysis of processes significantly affected by inhibition of IMD in progenitors. Column size indicates the degree of enrichment for each term, and dots indicate the log-transformed significance of the respective enrichment. (H) Representative sample of genes with affected expression upon inhibition of IMD in progenitors. See also Figures S1 and S2.

In a single-cell RNA sequencing profile of adult female *Drosophila* intestines, we identified 620 cells that expressed the progenitor-cell markers *esg*, *Dl*, and *N* (Figure S1). Within the progenitors, we also observed enriched expression of key IMD pathway components, including the PGN sensor *pgrp-lc*, the NF-kB transcription factor *relish*, and the IMD pathway target *pirk* (Figures 1A and 1B). Our data match earlier reports of IMD pathway gene expression in progenitors (Dutta et al., 2015; Hung et al., 2020) and raise the possibility that immune signals contribute to gut-progenitor-cell function.

To test IMD activity in progenitors, we used the *esgGAL4*, *GAL80*^*ts*^, *UASGFP* (*esg*^*ts*^) fly line to express a dominant inhibitory IMD protein (ImdD30A) in *Drosophila* progenitors (*esg*^*ts*^*/D30A*) for 10 days. Blocking IMD in progenitors did not stop infection-mediated expression of IMD-responsive antimicrobial peptides in enterocytes, demonstrating that inhibition of progenitor-cell IMD does not affect IMD activity in differentiated progeny (Figure S2). We then asked if blocking IMD had direct effects on the progenitor population. Both *esg*^*ts*^*/D30A* and control *esg*^*ts*^*/+* intestines had similar distributions of small, GFP-positive cells (Figure 1C) that rarely expressed the enteroendocrine cell marker *prospero* (Figure 1D), confirming that GFP exclusively marked progenitors in both lines. To measure effects of IMD on progenitors, we performed RNA sequencing (RNA-seq) analysis of gene expression in fluorescence-activated cell sorting (FACS)-purified GFP-positive cells from *esg*^*ts*^*/D30A* and *esg*^*ts*^*/+* flies (Figure 1E). We found that inactivation of IMD disrupted expression of 154 genes in progenitors (Figure 1F; Table S1), including key IMD pathway regulators (*pgrp-sd*, *pgrpsc1a*), glutathione metabolism genes required for detoxification of xenobiotic substances, and 24 genes known to respond to the commensal microbiome (Broderick et al., 2014) (Figures 1F–1H). Notably, the impacts of IMD inhibition extended beyond conventional antimicrobial responses and included diminished expression of genes associated with stem-cell growth and adhesion to the niche, such as the growth regulator *Xrp1*, the asymmetric cell division regulator *miranda* (*mira*), and the effector of extracellular matrix adhesion *Vinculin* (*Vinc*; Figures 1F–1H), suggesting potential growth-regulatory roles for IMD in progenitors.

### IMD modifies ISC division

**Figure 2:**
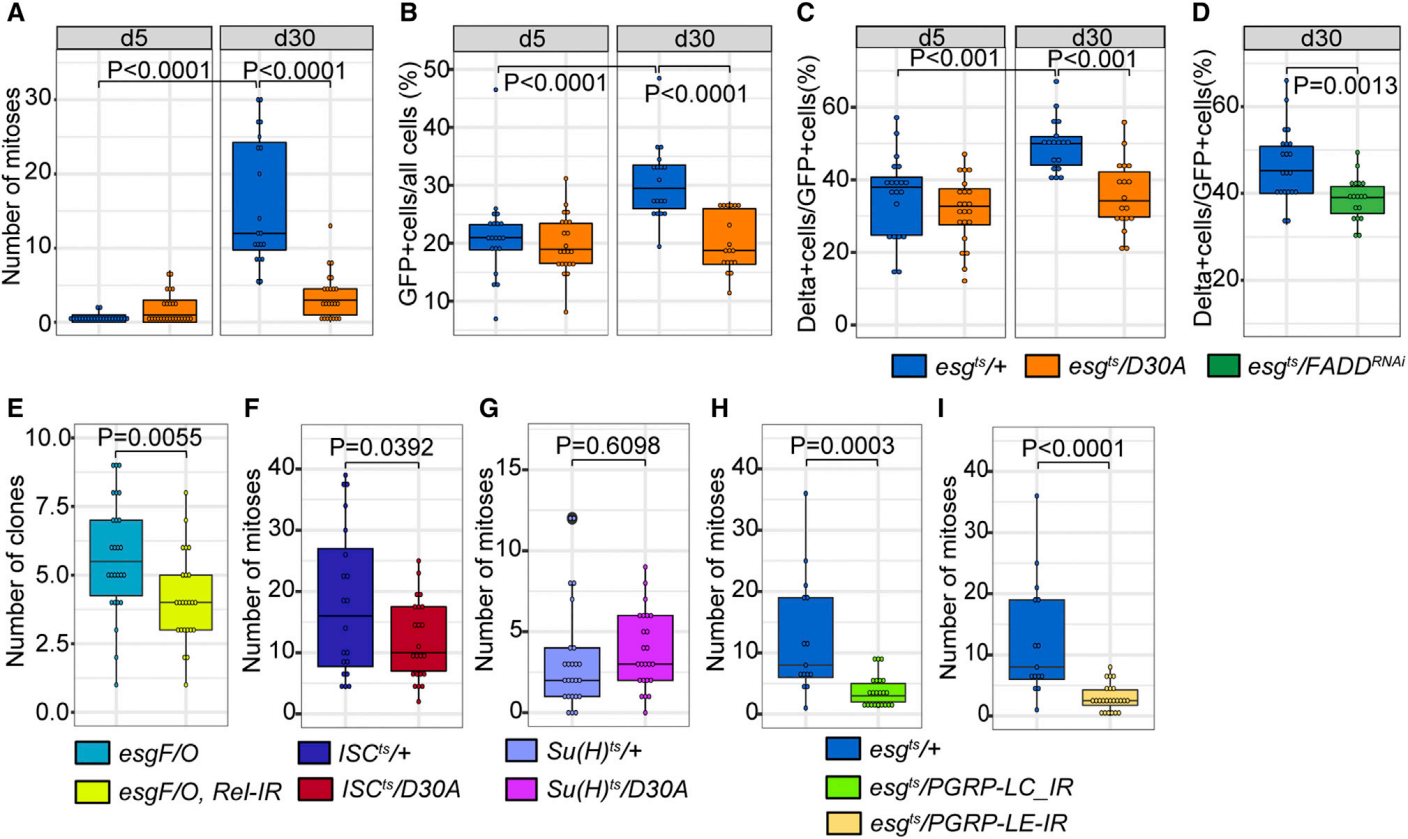
Progenitor-cell IMD activity modifies stem-cell proliferation (A) Quantification of mitoses per gut in intestines from *esg*^*ts*^/*+* and *esg*^*ts*^/*D30A* flies of the indicated ages. (B) Percentage of intestinal epithelial cells that express the progenitor marker *esg* in *esg*^*ts*^/*+* and *esg*^*ts*^/*D30A* flies. (C) Percentage of progenitors that express the stem-cell marker Delta in *esg*^*ts*^/*+* and *esg*^*ts*^/*D30A* flies. For (A)–(C), n = 20 at day 5 and 18 at day 30 in *esg*^*ts*^*/+* flies and n = 22 at day 5 and 18 at day 30 in *esg*^*ts*^/*D30A* flies. (D) Percentage of progenitors that express the stem-cell marker Delta in *esg*^*ts*^/*+* (n = 21) and *esg*^*ts*^/*FADD*^*RNAi*^ (n = 18) flies. (E) Quantification of GFP-marked mitotic clones in the posterior midgut of *esg*^*F/O*^ (n = 26) and *esg*^*F/O*^, *rel-IR* (n = 24) flies 9 days after marking of mitotic clones. (F) Quantification of mitoses per gut in *ISC*^*ts*^*/+* ( n = 21) and *ISC*^*ts*^*/D30A* (n = 24) 27-day-old flies. (G) Quantification of mitoses per gut in 27-day-old *Su(H)*^*ts*^/+ (n = 25) and *Su(H)*^*ts*^*/D30A* (n = 24) flies. (H and I) Quantification of mitoses per gut in 27-day-old *esg*^*ts*^/*+* (n = 15), *esg*^*ts*^/*PGRP-LC-IR* (n=22) (H) and *esg*^*ts*^/*PGRP-LE-IR* (n = 24) (I) flies. Statistical significance for (A)–(C) was calculated using an ANOVA followed by pairwise Tukey comparisons, and significance for (D)– (I) was calculated using a Student's t test.

As IMD inhibition affected the progenitor transcriptome, we asked if IMD also affects progenitor homeostasis. Specifically, we measured ISC mitoses by quantifying phospho-histone H3, the percentage of midgut epithelial cells that expressed the progenitor marker *esg*, and the percentage of progenitors that expressed the ISC marker *Delta* (*Dl*) in 5- and 30-day-old *esg*^*ts*^*/D30A* and *esg*^*ts*^*/+* intestines. In young *esg*^*ts*^*/+* intestines, we observed few ISC divisions (Figure 2A), approximately 20% *esg*+ cells in the posterior midgut (Figure 2B), and 40% Dl+ ISCs per progenitor (Figure 2C). Consistent with reports of age-related decline in gut function, we detected significantly increased numbers of ISC divisions, *esg*+ cells, and Dl+ cells in aged *esg*^*ts*^*/+* intestines relative to their 5-day-old counterparts (Figures 2A–2C). In contrast, we did not detect age-dependent increases in mitoses, progenitor numbers, or ISC numbers in 30-day-old *esg*^*ts*^*/D30A* intestines (Figures 2A–2C). Instead, 30-day-old *esg*^*ts*^*/D30A* intestines were characterized by significantly fewer mitoses, Dl+ ISCs, and progenitors than 30-day-old *esg*^*ts*^*/+* control flies. Importantly, these results are not an artifact of *imdD30A* expression, as progenitor-specific, RNAi-mediated depletion of the IMD pathway adaptor FADD caused a significant decline in the amounts of Dl-positive stem cells among intestinal progenitors of 30-day-old flies (Figure 2D). Furthermore, progenitor-specific inactivation of *relish* significantly impaired generation of mitotic clones in the posterior midgut (Figure 2E), confirming that genetic inhibition of IMD blocks intestinal epithelial proliferation. ISC-specific inactivation of IMD was sufficient to block proliferation (Figure 2F), whereas EB-specific inhibition of IMD had no effect (Figure 2G), suggesting a cell-autonomous role for IMD in controlling ISC division. Finally, we discovered that inhibition of the PGN sensors *PGRP-LC* or *PGRP-LE* (Figures 2H–2I) was sufficient to inhibit progenitor-cell proliferation. Collectively, our data indicate that inactivation of IMD in progenitors significantly impairs age-dependent accumulation of mitotically active progenitors in the adult midgut. We consider these findings particularly interesting as increased epithelial immune responses are a hallmark of the aging intestine (Broderick et al., 2014; Buchon et al., 2009a; Guo et al., 2014; Ren et al., 2007).

### Single-cell analysis uncovers impacts of progenitor-cell IMD on the intestinal epithelium

Similar to vertebrates, the *Drosophila* intestine is a highly heterogenous tissue. Multipotent stem cells generate distinct epithelial lineages that control nutrient acquisition, hormone production, and responses to intestinal microbes in a regionally specialized fashion. Thus, although our data implicate IMD in progenitor-cell division, we do not yet understand the consequences of blocking IMD in progenitors for the entire intestine. To determine the effects of progenitor-specific IMD inhibition on all epithelial cell types, we resolved the transcriptomes of 10-day-old *esg*^*ts*^/+ and *esg*^*ts*^*/D30A* intestines at the single-cell level (Figures S3A and S3B). After excluding dead cells and doublets, we prepared RNA sequencing profiles of 3,675 cells from *esg*^*ts*^/+ intestines and 3,654 cells from *esg*^*ts*^*/D30A* intestines. Using unsupervised graph-based clustering of data from *esg*^*ts*^/+ intestines, we identified all cell types previously described in the adult gut, including progenitors that expressed growth and differentiation regulators, enteroendocrine cells that produced peptide hormones, and enterocytes dedicated to digestion (Figure S3). A more detailed examination of single-cell transcriptomes from *esg*^*ts*^/+ intestines uncovered clear signs of specialization among the individual cell types. Specifically, we discovered regionalized and cell-type-specific expression patterns for regulators of metabolism, growth, differentiation, PGN sensing and scavenging, and oxidative stress responses (Figure S4). Thus, our profile of *esg*^*ts*^/+ guts accurately recapitulated known features of spatial and functional specialization within the fly gut (Dutta et al., 2015; Hung et al., 2020), providing a reliable control for analysis of intestines with impaired progenitor-cell IMD.

**Figure 3:**
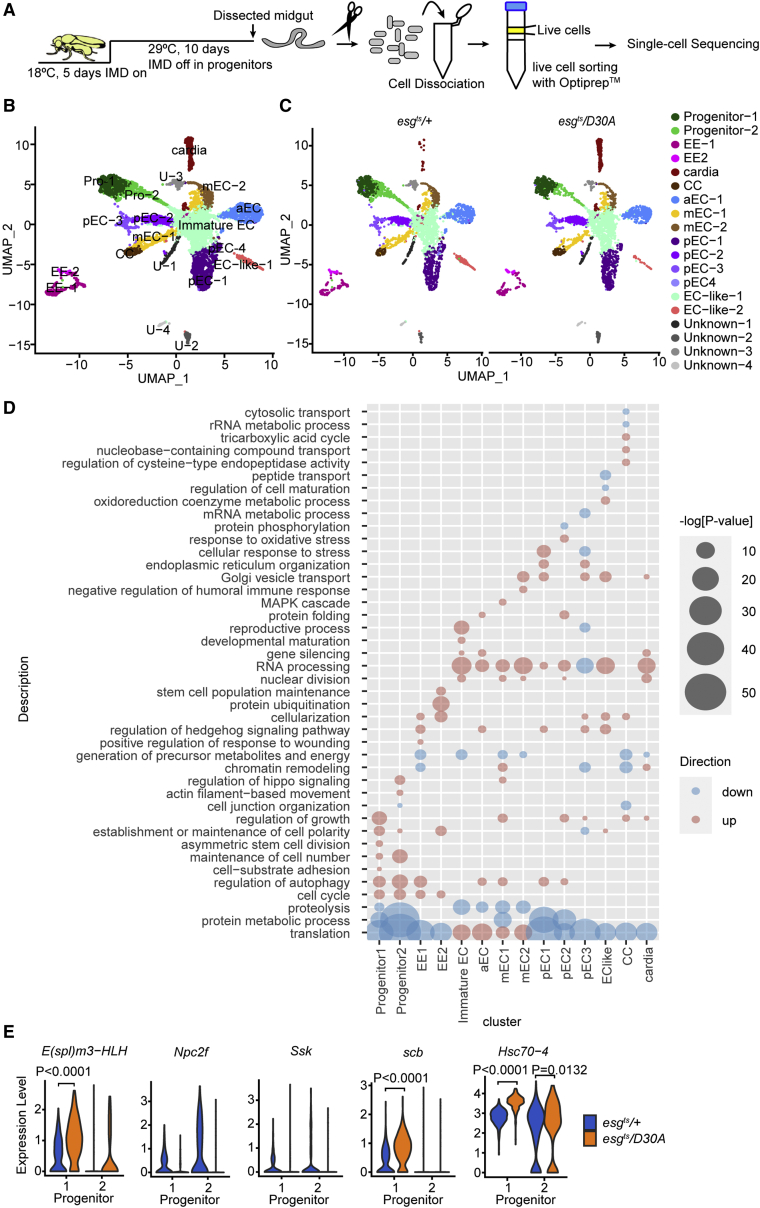
Inactivating IMD in progenitors affects transcriptional activity in all intestinal epithelial cell types (A) Schematic representation of an experimental strategy for single-cell transcriptomic analysis of purified intestinal epithelial cells from *esg*^*ts*^/*+* and *esg*^*ts*^/*D30A* flies. (B) UMAP plot visualizing cell types in integrated data from *esg*^*ts*^/*+* and *esg*^*ts*^/*D30A* flies based on the expression of marker genes. Cells are color-coded by cell type. (C) The same data from (B), split into the labeled genotypes. EE, enteroendocrine cells; CC, copper cells; EC, enterocytes subdivided according to anterior-posterior distribution along the intestine (a, anterior; m, middle; p, posterior). (D) Gene Ontology term analysis of cell-type-specific processes significantly affected by progenitor-restricted inhibition of IMD. Bubble size indicates the log-transformed significance of the respective enrichments. Pink bubbles indicate enhanced terms, and blue bubbles indicate underrepresented terms. (E) Representative violin plots of expression levels for the indicated genes in progenitors of *esg*^*ts*^/*+* and *esg*^*ts*^/*D30A* flies. p values indicate significantly different expression levels. For *Npc2f* and *Snakeskin* (*Ssk*), no expression was observed in progenitors of *esg*^*ts*^/*D30A* flies. See also Figures S3 and S4.

To determine if blocking IMD in progenitors affects mature epithelial cells, we used the integrated data analysis workflow in Seurat to identify cell-type-specific differences in gene-expression patterns between *esg*^*ts*^/+ and *esg*^*ts*^*/D30A* intestines. Unsupervised clustering of the integrated data resolved progenitors, enterocytes, and enteroendocrine cells, as well as cardia, copper cells, an enterocyte-like cluster, a cluster of immature enterocytes, and three lineages of unknown function (Figure 3B). Comparisons between the two genotypes suggested mild effects of blocking IMD in progenitors on the generation of mature IECs. For example, we noted fewer enterocyte (EC)-like cells and considerably more cardia in intestines from *esg*^*ts*^*/D30A* than in *esg*^*ts*^*/+* flies (Figure 3B). Furthermore, blocking IMD in progenitors affected the expression of genes associated with critical regulatory functions in the gut. For example, intestines from *esg*^*ts*^*/D30A* flies were characterized by shifts in RNA processing and translation in ECs, diminished precursor metabolite generation in enteroendocrine cells, and increased expression of genes involved in autophagy, cell polarity, and adhesion in progenitors (Figure 3C). Notably, blocking IMD in progenitors did not affect expression of antimicrobial peptides, or PGN recognition proteins, in differentiated ECs (Table S2), further arguing that expression of *imdD30A* in progenitor cells does not inhibit immune activity in progeny. Instead, we observed substantial effects of inhibiting IMD on expression of genes with essential roles in progenitor-cell division and polarity (Figure 3D), including the Notch signaling modifier *Npc2f*, the Notch pathway target *E*(*spl*)*m3-HLH*, and *Snakeskin* (*Ssk*) a key regulator of intestinal-stem-cell activity (Figure 3E). Thus, and consistent with data presented in Figures 1 and 2, our results indicate that inhibition of IMD in progenitors has significant effects on progenitor-cell homeostasis.

As we believe our gene expression data are likely of value to the community outside the scope of the current study, we have deposited both sets on the Broad Institute Single Cell Portal (see experimental procedures for further details).

### IMD affects developmental trajectories within the progenitor compartment

**Figure 4:**
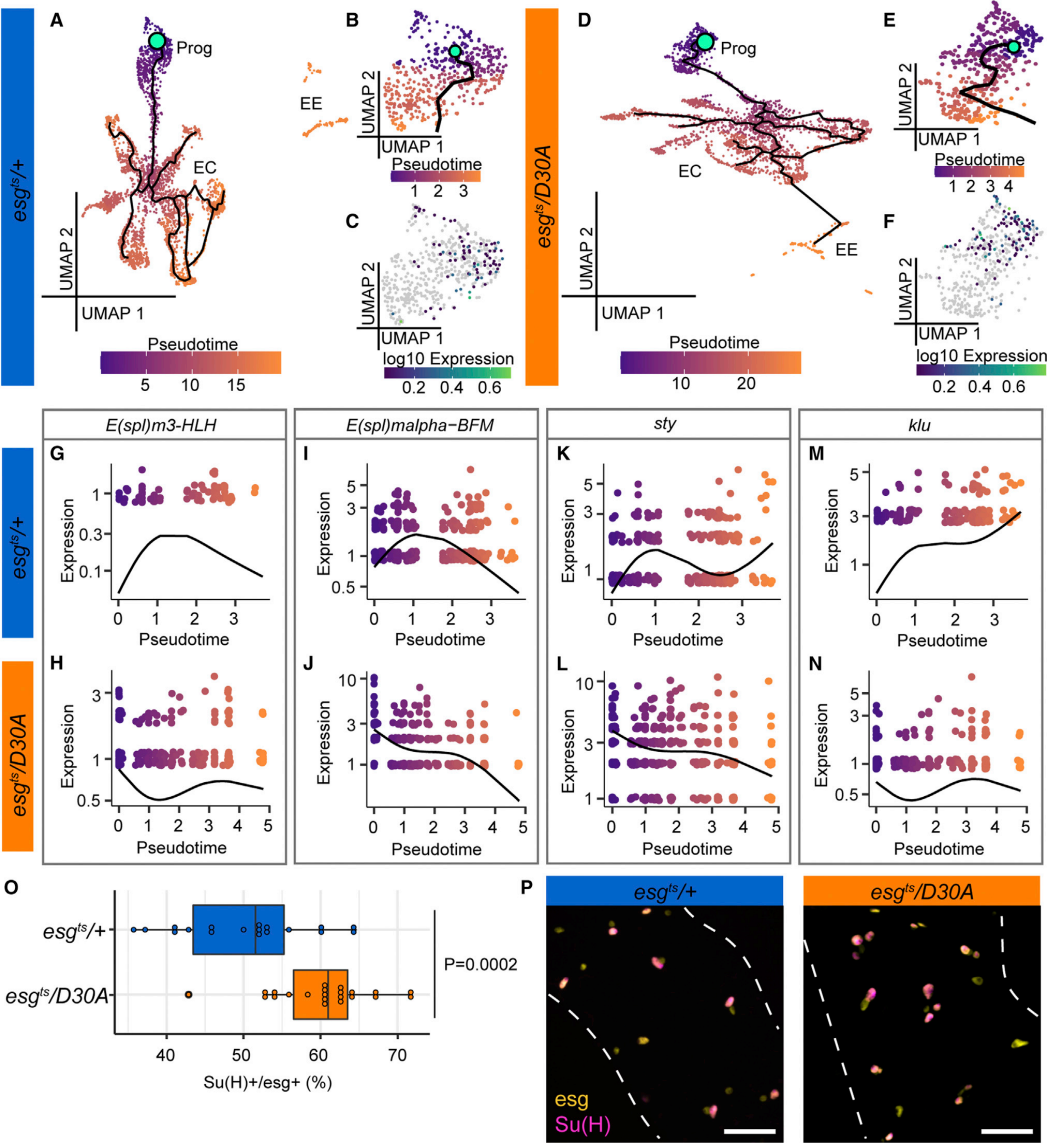
Inhibition of IMD affects developmental trajectories within the progenitor compartment Single-cell datasets from Seurat for each individual genotype were loaded into Monocle3, and pseudotime analysis was performed on midgut epithelial cells. (A and D) *esg*^*ts*^*/+* midguts with wild-type progenitors (A) and *esg*^*ts*^*/D30A* midguts with IMD-deficient progenitors (D). Mint green circles denote the root node and beginning of the intestinal trajectories. Dark purple marks cells at the beginning of pseudotime, while orange marks cells late in pseudotime. Black lines show trajectories. Prog, progenitors; EC, enterocytes; EE, enteroendocrine cells. (B and E) Pseudotime within progenitor subsets of (A) and (D), respectively. (C and F) *Delta* (*Dl*) expression patterns within *esg*^*ts*^*/+* progenitors (C) and *esg*^*ts*^*/D30A* progenitors (F). Gray dots are cells with no detectable expression. (G–N) Expression of Notch target genes *E(spl)m3-HLH*, *E(spl)malpha-BFM,* the EGF inhibitor *sprouty (sty*), and the EC fate regulator *klumpfuss* (*klu*) over pseudotime within progenitor subsets of the indicated genotypes. (O) Percent of *esg+* progenitors that are positive for the enteroblast marker *Su(H)+* in *esg*^*ts*^*, UAS-CFP, Su(H)-GFP/+* (n = 18) and *esg*^*ts*^*, UAS-CFP, Su(H)-GFP /D30A* (n = 22) posterior midguts 14 days after transgene expression. Significance found using Student’s t test. (P) Representative images of intestines used to gather data for (O). Scale bars represent 25 μm. See also Figure S5.

We were intrigued by our observation that blocking IMD in progenitors significantly affected expression of genes required for progenitor-niche interactions and progenitor differentiation. Therefore, we used Monocle to prepare pseudotime developmental trajectories for *esg*^*ts*^*/+* and *esg*^*ts*^*/D30A* intestines. Analysis of the respective datasets successfully re-created developmental transitions from multipotent progenitors to differentiated lineages in both lines (Figures 4A and 4D). To directly examine effects of IMD on progenitors, we created subsets of the progenitor population for each genotype and analyzed gene expression in pseudotime for the respective subsets. Examination of the progenitor population from both genotypes revealed gene-expression patterns characteristic of developmental transitions along a pseudotime trajectory (Figures 4B and 4E). For example, both progenitor populations were characterized by expression of the ISC marker *Dl* in early stages of pseudotime (Figures 4C and 4F). However, IMD inhibition resulted in premature and prolonged pseudotime expression of the Notch targets *E(spl)m3-HLH* (Figures 4G and 4H) and *E(spl)malpha-BFM* (Figures 4I and 4J), the EGF regulator *sprouty* (*sty*; Figures 4K and 4L), the EC fate regulator *klumpfuss* (*klu*; Figures 4M and 4N), and numerous markers of EC maturation (Figure S5), indicating effects of progenitor-cell IMD on EC differentiation. To test if blocking IMD in progenitors impacts the transition from ISC to enteroblast, we monitored expression of fluorescent markers in *esgGAL4*, *UAS-CFP*, and *Su(H)-GFP; GAL80*^*ts*^ flies that expressed ImdD30A. In these flies, ISCs are visible as CFP-positive cells (pseudocolored as yellow), and enteroblasts are visible as CFP and GFP double-positive cells (pseudocolored as magenta). Consistent with putative interactions between IMD and Notch, we found that blocking IMD significantly increased the percentage of progenitors that expressed the enteroblast marker *Su*(*H*)*-GFP* (Figures 4O and 4P). Thus, in agreement with the loss of ISCs noted in *esg*^*ts*^*/D30A* intestines (Figure 2), our data argue that IMD activity influences progenitor-cell composition in the fly intestinal epithelium.

### Progenitor IMD affects generation of mature enteroendocrine cells

**Figure 5:**
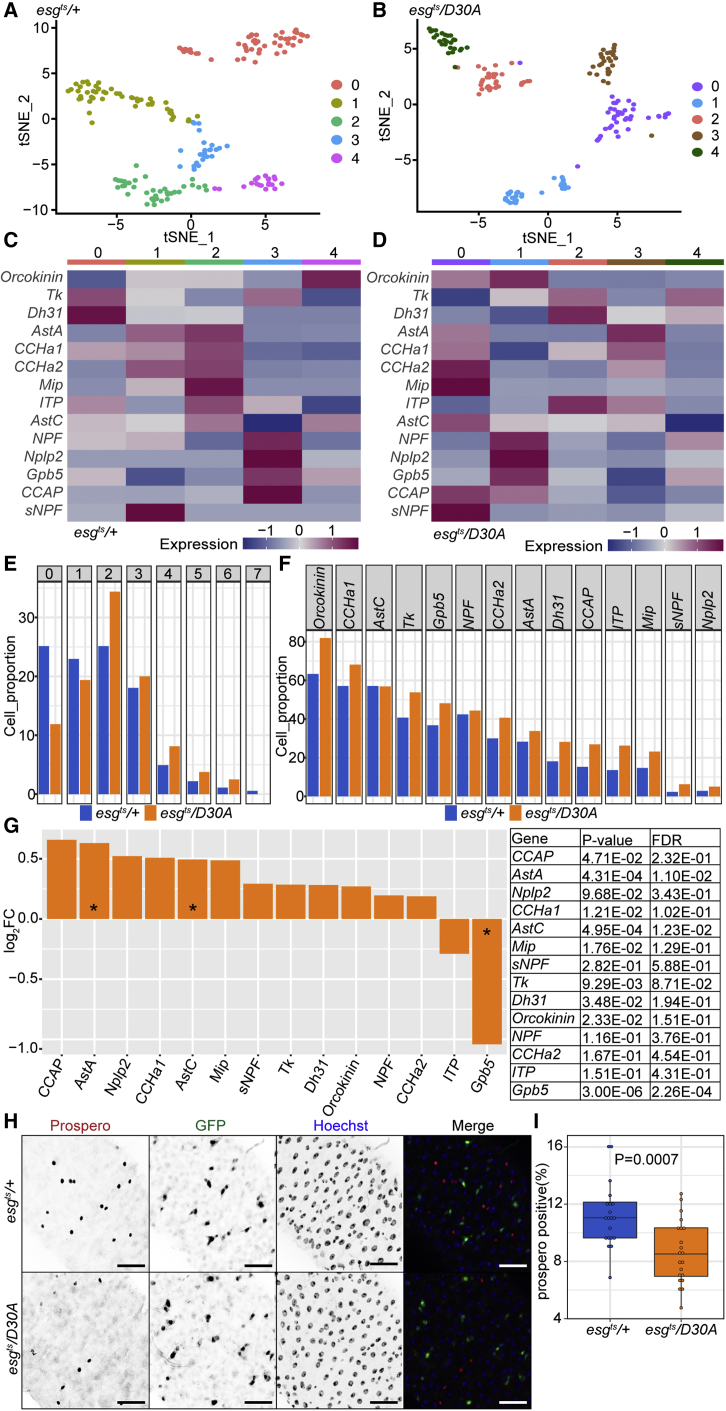
Inhibition of progenitor-cell IMD affects generation of mature enteroendocrine cells (A and B) t-distributed stochastic neighbor embedding (tSNE) plot visualizing subsets of *prospero*-positive enteroendocrine cells in *esg*^*ts*^/*+* (A) and *esg*^*ts*^/*D30A* intestines (B) based on the expression of marker genes. Cells are color-coded by cell subset. (C and D) Heatmap showing relative expression of fourteen peptide hormones in each enteroendocrine cell subset in *esg*^*ts*^/*+* (C) and *esg*^*ts*^/*D30A* (D) intestines. (E) Quantification of the percentage of enteroendocrine cells that express the indicated numbers of peptide hormones. Genotypes are color-coded as indicated. (F) Quantification of the percentage of enteroendocrine cells that express the indicated peptide hormone. Genotypes are color-coded as indicated. (G) Quantification of the relative expression of each peptide hormone in isolated posterior midguts from *esg*^*ts*^/*D30A* flies relative to *esg*^*ts*^/*+* flies based on bulk RNA-seq analysis. (H) Visualization of Prospero, GFP, and DNA (Hoechst) in intestines of 10-day-old *esg*^*ts*^/*+* and *esg*^*ts*^/*D30A* flies. Scale bars represent 25 μm. (I) Quantification of the percentage of intestinal epithelial cells that express the enteroendocrine cell marker *prospero* in the intestines of 10-day-old flies as indicated. Statistical significance was calculated using Student’s t test. For *esg*^*ts*^/*+* flies n = 20, and for *esg*^*ts*^/*D30A* flies n = 21.

IMD inhibition impaired ISC proliferation, diminished ISC numbers, and impacted cell-type composition within the progenitor compartment, suggesting possible effects of progenitor-cell IMD on the development of mature epithelial cells. To determine if inhibition of IMD in progenitors affects epithelial differentiation, we monitored the *prospero*-positive enteroendocrine (EE) cell population in *esg*^*ts*^*/+* and *esg*^*ts*^*/D30A* intestines. We focused on EE cells, as fly EE cells have been characterized to a single-cell resolution (Guo et al., 2019; Hung et al., 2020), permitting detailed comparisons between *esg*^*ts*^*/+* and *esg*^*ts*^*/D30A* intestines. *Drosophila* EE cells can be divided into subsets with distinct peptide-hormone expression profiles that are stable during homeostasis or after recovery from infection (Beehler-Evans and Micchelli, 2015). Therefore, we tested if inhibition of IMD in progenitors affected the representation of EE subsets in the intestine. Using unsupervised clustering, we found that EE cells clustered into five subsets in both genotypes (Figures 5A and 5B). In both genotypes, each EE subset had a signature hormone-expression pattern (Figures 5C and 5D). In some cases, subset-restricted expression patterns were conserved between *esg*^*ts*^*/D30A* and *esg*^*ts*^*/+* EE cells. For example, matching an earlier characterization of EE subsets (Beehler-Evans and Micchelli, 2015), we found that cells from *esg*^*ts*^*/+* subset zero, and from *esg*^*ts*^*/D30A* subset two, expressed *Tk* and *Dh31*. Likewise, *esg*^*ts*^*/+* subset three cells and *esg*^*ts*^*/D30A* subset one cells were characterized by enhanced expression of *NPF* and by partial expression of *Gbp5* and *CCAP*. In contrast, we did not detect a counterpart of *esg*^*ts*^*/D30A* subset zero cells in *esg*^*ts*^*/+* controls, and we saw minimal conservation of *esg*^*ts*^*/+* subset zero gene expression patterns in *esg*^*ts*^*/D30A* EE cells, suggesting functional differences between EE cells in *esg*^*ts*^*/D30A* flies compared with *esg*^*ts*^*/+* flies. When we classified EE cells based on the number of peptides they expressed, we noted further differences between *esg*^*ts*^*/+* and *esg*^*ts*^*/D30A* intestines. In particular, fewer EE cells expressed zero or one peptide in *esg*^*ts*^*/D30A* guts than in *esg*^*ts*^*/+* guts, and a greater proportion expressed two or more peptides (Figure 5E). Likewise, for thirteen of fourteen peptides examined, a greater percentage of *esg*^*ts*^*/D30A* EE cells expressed the respective peptide than *esg*^*ts*^*/+* controls (Figure 5F), indicating enhanced peptide expression in *esg*^*ts*^*/D30A* flies. To directly test the effects of blocking IMD in progenitors on peptide-expression levels, we performed an RNA-seq analysis of dissected midguts from *esg*^*ts*^*/D30A* and *esg*^*ts*^*/+* flies. With the exceptions of *ITP* and *Gpb5*, we found that blocking IMD in progenitors resulted in increased expression of the remaining twelve peptides (Figure 5G), confirming a link between IMD inhibition and peptide-hormone expression. Finally, we quantified EE numbers in posterior midguts of *esg*^*ts*^*/+* and *esg*^*ts*^*/D30A* flies. We found that inhibition of IMD in progenitors decreased the proportion of mature EE cells by roughly 20% relative to *esg*^*ts*^*/+* controls (Figure 5H). Combined, our results establish that inhibition of progenitor-cell IMD disrupts peptide-hormone expression patterns in mature EE cells, decreases the amount of total EE cells, and increases the expression of most peptide hormones, confirming a link between progenitor-cell IMD activity and EE-cell development.

## Discussion

Notch, BMP, and WNT pathways regulate intestinal progenitor-cell growth and differentiation in vertebrates and invertebrates (Barker, 2014; Casali and Batlle, 2009; Miguel-Aliaga et al., 2018; Sancho et al., 2015; Vooijs et al., 2011; Xu et al., 2011). In contrast, it is less clear what effects progenitor-specific activation of germline-encoded immune responses has on epithelial homeostasis. Several studies indicated survival and growth-regulatory effects of host immunity on intestinal progenitors. For example, the PGN receptor NOD2 is enriched in ISCs of mice (Nigro et al., 2014) and protects ISCs from irradiation-induced cytotoxicity (Levy et al., 2020), while mutations in NOD2 are linked to Crohn’s disease (Hugot et al., 2001; Ogura et al., 2001). Likewise, TLR4 is expressed to higher levels in intestinal crypts (Price et al., 2018), where its activation promotes apoptosis and inhibits proliferation (Naito et al., 2017; Neal et al., 2012). In contrast, epithelium-wide activation of TLR4 promotes epithelial repair by activating EGF and JAK/STAT pathways in mice challenged with dextran sulfate sodium (Fukata et al., 2006; Hsu et al., 2010). These studies support roles for immune pathways in proliferative responses to extrinsic challenges. However, we do not know if progenitor-specific immune activity impacts homeostatic growth and differentiation. We consider this an important question, as intestines contain dense microbial communities that promote growth and influence cell-fate choices in intestinal epithelia (Ferguson and Foley, 2021).

We measured growth and differentiation in adult *Drosophila* midguts that we engineered to lack IMD activity in progenitors. The IMD pathway is highly similar to mammalian tumor necrosis factor receptor signaling, and IMD exerts broad regulatory effects on intestinal transcription (Broderick et al., 2014). In flies, IMD has context-dependent effects on ISC proliferation. IMD-pathway mutants have elevated rates of mitoses that are driven by the microbiome (Buchon et al., 2009b; Guo et al., 2014; Paredes et al., 2011), but IMD is not required for the proliferative burst observed after challenges with *Ecc15* (Zhai et al., 2018). In contrast, infection with *Vibrio cholerae* blocks ISC proliferation in an IMD-dependent manner (Fast et al., 2020; Wang et al., 2013), whereas *Herpetomonas muscarum* induces IMD-dependent proliferation (Wang et al., 2019). In the absence of infection, persistent activation of IMD in progenitors increases ISC division frequency and skews differentiation towards elevated numbers of EE cells (Petkau et al., 2017). Similarly, overexpression of PGRP-LC in ECs induces Rel-dependent proliferation (Zhai et al., 2018). There are conflicting data on consequences of IMD inactivation in progenitors, with one study suggesting decreased proliferation (Wang et al., 2019), and a separate study indicating increased proliferation (Wang et al., 2013). We found that progenitor-specific inactivation of IMD diminished ISC proliferation, impaired age-dependent accumulation of *esg*-positive and Dl-positive progenitors, elevated the number of *Su*(*H*)-positive enteroblasts, and resulted in differentiation defects that included both fewer EE cells and shifts in gene-expression patterns associated with EC subtypes. Thus, our work suggests that progenitor-specific immunity contributes to epithelial homeostasis in flies and raises several questions about effects of progenitor-cell IMD on the adult gut.

Which progenitor cell requires IMD to regulate differentiation? The fly progenitor compartment consists of undifferentiated ISCs and post-mitotic enteroblasts that are committed to EC cell fate. Progenitors express IMD-pathway components, and genomic studies, including data presented here, show that ISCs and enteroblasts have highly similar gene-expression profiles (Dutta et al., 2015; Hung et al., 2020), suggesting that both cell types are likely equally competent at IMD activation. ISCs are basally situated within the midgut epithelium and are not expected to make frequent, direct contacts with the intestinal lumen. Enteroblasts are the apical daughters of ISC divisions that occur at oblique angles to the basement membrane. Thus, it seems more plausible that enteroblasts directly contact the lumen where they can detect PGN. However, it is important to note that gut-derived PGN is not strictly confined to the intestinal lumen in flies or vertebrates. PGN crosses the epithelial barrier, even in the absence of detectable breaches, and several mechanisms are in place to prevent accumulation of PGN in the fly hemolymph (Capo et al., 2017; Gendrin et al., 2009; Paredes et al., 2011; Troha et al., 2019; Zaidman-Rémy et al., 2006). Thus, we cannot exclude the possibility that passive, or active, transport mechanisms allow diffusion of PGN across the gut barrier to ISCs, which in turn activate IMD and modulate differentiation responses in the hosts. In this regard, we consider it interesting that several vertebrate pattern-recognition receptors have cell-type-specific, apicobasal distribution patterns. For instance, TLR4 is enriched apically in villi and basolaterally in crypts of the human colon (Fusunyan et al., 2001). Furthermore, apical stimulation of TLR9 promotes JNK activation, whereas basolateral stimulation of TLR9 leads to NF-kB activation, and IL-8 production (Lee et al., 2006). In future assays, it will be interesting to determine if basolateral detection of PGN influences fate choices within the fly progenitor compartment.

Is progenitor-specific IMD necessary to generate growth-regulatory ECs? Inhibition of progenitor-cell IMD did not block IMD-dependent immune responses in ECs, confirming that the phenotypes reported here are not a consequence of ImdD30A perdurance in differentiated epithelia. However, we also found that inhibition of IMD in progenitors had consequences for epithelial differentiation, including effects on gene-expression patterns within mature ECs. As ECs produce paracrine regulators of progenitor proliferation and differentiation, we cannot exclude the possibility that blocking IMD in progenitors disrupts enteroblast differentiation in a manner that modifies the ability of ECs to transduce growth and differentiation cues to progenitors. This may be particularly important in the context of epithelial damage, where secreted factors from dying ECs accelerate ISC proliferation to maintain the epithelial barrier and regenerate a mature gut. In this scenario, IMD activity in progenitors is important to establish homeostatic intercellular communications between ECs and the progenitor compartment, and loss of progenitor-cell IMD interrupts a developmental loop between progenitors and ECs. Consistent with requirements for IMD in the control of epithelial differentiation, we noticed that IMD in progenitors affected the generation of mature EE cells. Specifically, inhibition of progenitor-cell IMD led to a decline in EE numbers but a general increase in the expression of peptide hormones, possibly as a compensatory mechanism. Notably, flies raised in an axenic environment have fewer enteroblasts and more EE cells (Broderick et al., 2014), indicating non-overlapping contributions of progenitor-cell immunity and gut microbes to developmental trajectories of ISCs.

Does IMD have regional effects on intestinal progenitors? Intestines are functionally specialized along the rostro-caudal axis, with distinct partitions governing various aspects of food digestion and absorption. IMD also displays clear signs of regional specialization. The foregut is characterized by enriched expression of PGRP-LC, and Rel regulates expression of chitin-binding proteins that contribute to peritrophic matrix construction (Buchon et al., 2009b; Neyen et al., 2012). In the midgut, PGN detection primarily relies on PGRP-LE, particularly in the posterior midgut (Bosco-Drayon et al., 2012; Neyen et al., 2012). Activation of IMD in the anterior midgut results in expression of antimicrobial peptides that protect the fly from ingested microbes (Buchon et al., 2013b). In contrast, IMD activation leads to delamination of damaged cells in the midgut of flies infected with pathogenic bacteria, most notably in the R4 region of the posterior midgut (Zhai et al., 2018). In the posterior midgut, the transcription factor caudal prevents IMD-dependent antimicrobial peptide expression (Ryu et al., 2008). Instead, IMD induces expression of molecules that dampen immune signaling, including the PGRP-LC inhibitor *pirk*, and amidases that scavenge PGN (Bosco-Drayon et al., 2012). As a result, posterior midgut IMD establishes a tolerogenic environment for commensal bacteria. Notably, loss of IMD pathway inhibitors or expression of PGRP-LC in ECs, increases proliferation in the posterior midgut (Paredes et al., 2011; Zhai et al., 2018), raising the possibility that suppression of posterior midgut IMD activity is required to prevent excess proliferation in the absence of infection. With a large collection of genetic reagents, and accessible genomic methods, the fly is an excellent system to systematically characterize regional effects of immune responses on progenitor-cell function. Given the evolutionary conservation of immune responses, we believe the findings reported in this study to be of relevance for understanding fundamental principles of immune-regulated intestinal homeostasis.

## Experimental procedures

### Fly husbandry

Flies were raised on corn meal medium (Nutri-Fly Bloomington Formulation, https://bdsc.indiana.edu/information/recipes/bloomfood.html; Genesse Scientific) at 18°C or 25°C. All experimental flies were adult virgin females kept under a 12h:12h light:dark cycle and maintained at 18°C during collection then shifted to 29°C to express downstream genes as indicated. We used *w*^*1118*^ as a wild-type strain, backcrossed *UAS-imdD30A* transgenic lines into the *w*^*1118*^ background for eight generations prior to use, and used standard husbandry methods to ensure that *esg*^*ts*^ (*esg-GAL4*, *tub-GAL80*^*ts*^, *UAS-GFP*) flies had the same first and third chromosomes as our *w*^*1118*^ line. Fly lines used in this study were: *w;esg-GAL4,tubGAL80*^*ts*^*,UAS-GFP* (referred to as *esg*^*ts*^); *UAS-FADD*^*RNAi*^ (VDRC ID# 7926); *w*^*1118*^ (VDRC ID# 60000); *w;esg-GAL4,UAS-CFP, Su(H)-GFP;tubGal80*^*ts*^ (*esg*^*ts*^*,UAS-CFP,Su(H)-GFP*); *GS 5961* (Mathur et al., 2010); *dpt-GFP*, *esg-GAL4, tubGAL80ts, UAS-GFP;UAS-flp, Act>CD2>GAL4* (referred to as *esg*^*F/O*^); *Esg[ts], Su(H) Gal80* (referred to as *ISC*^*ts*^); *Su(H)GBE-Gal4*^*ts*^ (referred to as *Su(H)*^*ts*^); *PGRP-LE RNAi* (VDRC ID# 108199); *PGRP-LC RNAi* (VDRC ID# 101636); *Rel-RNAi* (VDRC ID# 49413); and *40D-UAS* (control for VDRC KK lines, VDRC ID# 60101) . To induce GFP-marked mitotic clones using the *esg*^*F/O*^ system, flies of the indicated genotype were raised at 18°C for 3 days after eclosion, shifted to 29°C for 16 h, then raised at 25°C for an additional 9 days.

### Data availability

The accession number for the gene expression data reported in this paper is GEO: SuperSeries GSE141897 (GSE171001 and GSE141896). The accession number for the Single cell gene expression data reported in this paper is Broad Institute Single Cell Portal: for *esg*^*ts/+*^ flies (https://singlecell.broadinstitute.org/single_cell/study/SCP1696/single-cell-expression-data-for-d-melanogasterwild-type-intestines) and for *esg*^*ts/D30A*^ flies (https://singlecell.broadinstitute.org/single_cell/study/SCP1699/single-cell-expression-data-for-d-melanogasterintestines-with-immune-deficient-progenitor-cells#study-summary).

## Author contributions

Conceptualization: M.S. and E.F.; methodology: M.S. and E.F.; formal analysis: M.S., M.F., and E.F.; investigation: M.S., M.F., R.J.W., L.O.J., and K.P.; data curation: M.S. and M.F.; writing – original draft: M.S. and E.F.; writing – review & editing: M.S., M.F., and E.F.; visualization: M.S. and M.F.; supervision: E.F.; project administration: E.F.; funding acquisition: E.F.

## Conflicts of interest

The authors declare no competing interests.

## Supplemental information

Document S1. Supplemental experimental procedures, Figures S1–S5, and Table S2

Table S1. Inactivation of IMD disrupted expression of 154 genes in progenitors, related to Figure 1

Document S2. Article plus supplemental information
