# Supplementary material for: Immune regulation of intestinal-stem-cell function in Drosophila
Source: Stem Cell Reports. 2022 Mar 17;17(4):741–55. doi: 10.1016/j.stemcr.2022.02.009 (PMC9023782; doi:10.1016/j.stemcr.2022.02.009)
Supplement: Document S1. Supplemental experimental procedures, Figures S1–S5, and Table S2 [file mmc1.pdf]

Stem Cell Reports, Volume *17*

Supplemental Information

Immune regulation of intestinal-stem-cell function in *Drosophila*

Minjeong Shin, Meghan Ferguson, Reegan J. Willms, Lena O. Jones, Kristina Petkau, and Edan Foley

SUPPLEMENTAL FIGURES

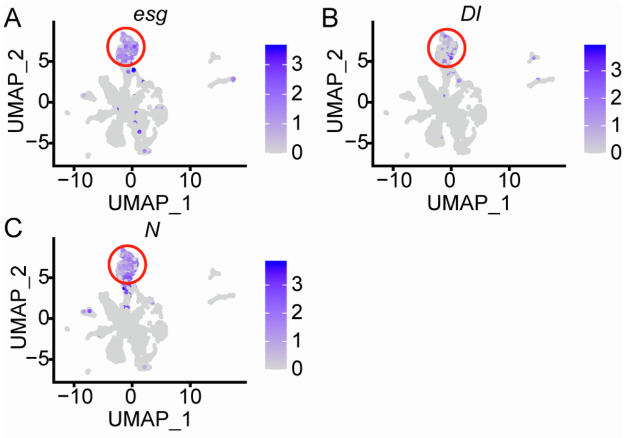

Figure S1

Figure S1. **Expression pattern of *Drosophila* progenitor cell markers, Related to Figure 1.**
Feature plots showing expression of *Drosophila* progenitor cell markers *escargot* (*esg*), *Delta (Dl*), and *Notch* (*N*) in an unsupervised UMAP prepared with single-cell expression data from adult *esg*^{ts}/+ female *Drosophila* midguts. Progenitor cells are indicated with a red circle.

A

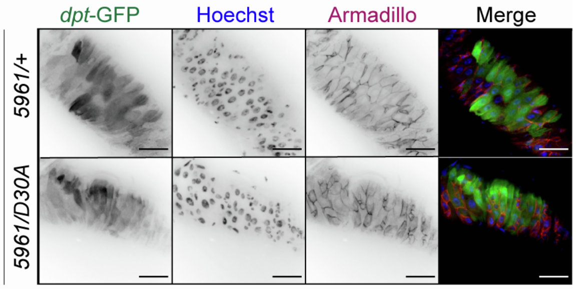

B

Genotype	Control	D30A
Sample size	9	10
% GFP +ive	100	100

Control: *GS 5961/+;dpt-GFP*

D30A: *GS 5961/D30A;dpt-GFP*

Supplementary Figure 2

Figure S2. Inhibition of IMD in progenitor cell does not affect IMD activity in differentiated progeny, related to Figure 1.

A: Visualization of the IMD pathway reporter *diptericin:GFP* (*dpt*-GFP), DNA (Hoechst), and the beta-catenin ortholog Armadillo in intestines of adult female *Drosophila* infected overnight with pathogenic *Ecc15*. The *esg^{ts}* line used in the rest of the study marks progenitor cells with GFP, preventing us from unambiguously identifying cells that expressed GFP under control of the *dpt* promoter in an *esg^{ts}* background. Therefore, we used the *GS5961* gene switch fly line for RU486-dependent induction of the GAL4 transcription factor in intestinal progenitor cells in this experiment. In the upper row, we visualized *dpt*-GFP expression in *GS5961/+* (*5961/+*) flies, and in the lower row, we visualized *dpt*-GFP expression in *GS5961/UAS-imdD30A* (*5961/D30A*) flies. Both lines were treated with RU486 for 48h prior to infection. Scale bars represent 25μm. **B:** Quantification of fly guts of the indicated genotypes that expressed *dpt*-GFP in enterocytes after infection with *Ecc15.* Expression of imdD30A in progenitors (*D30A*) did not prevent infection-mediated activation of IMD responses in mature enterocytes.

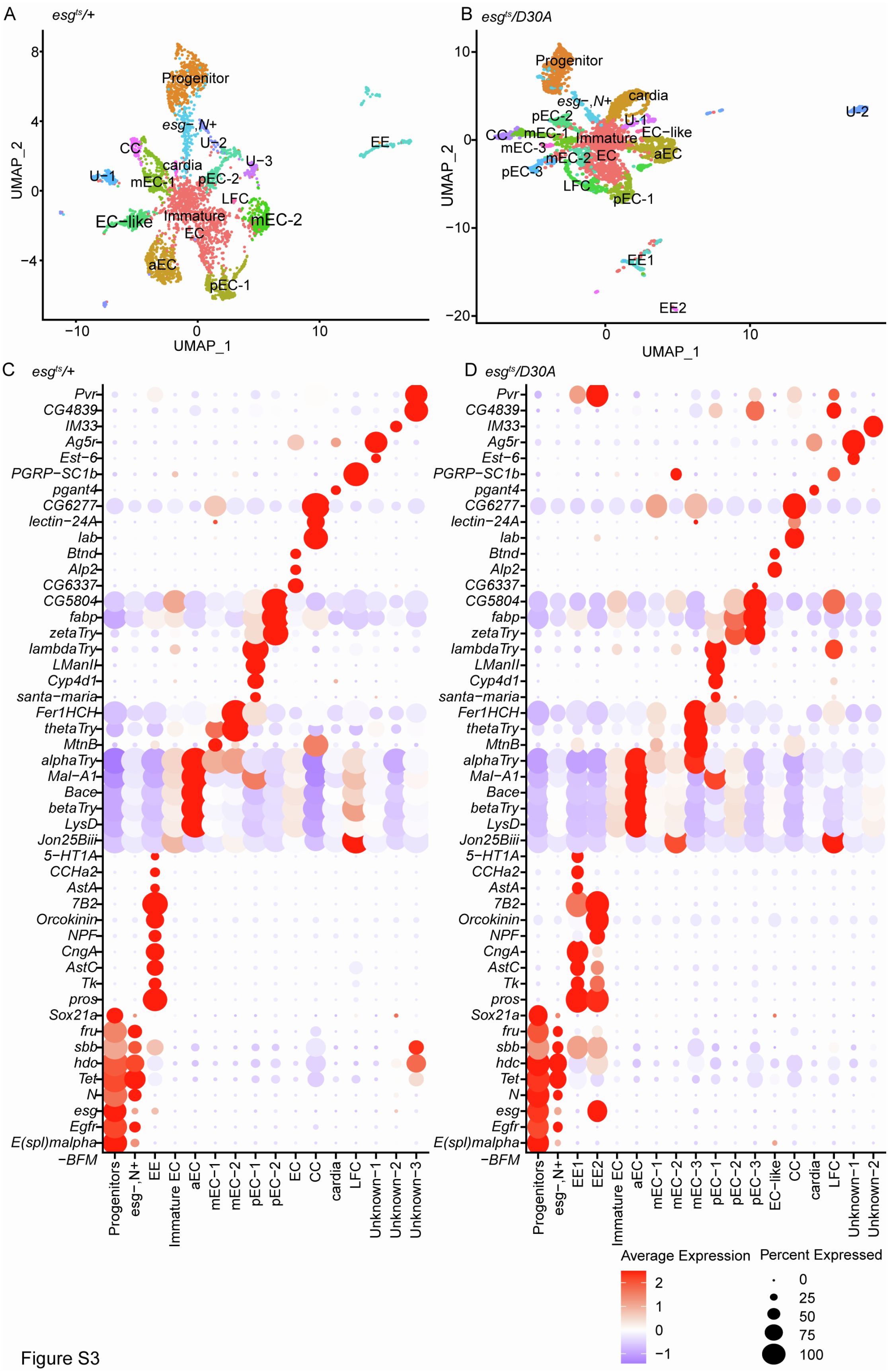

A
esgts/+
Progenitor
esg−,N+
U-2
CC
cardia
U-3
EE
U-1
mEC-1
pEC-2
LFC
EC−like
Immature EC
mEC-2
aEC
pEC-1
UMAP_1
UMAP_2
B
esgts/D30A
Progenitor
esg−,N+
cardia
pEC-2
U-1
U-2
CC
mEC-1
Immature
EC−like
mEC-3
mEC-2
EC
aEC
pEC-3
LFC
pEC-1
EE1
EE2
C
esgts/+
D
esgts/D30A
Pvr
CG4839
IM33
Ag5r
Est−6
PGRP−SC1b
pgant4
CG6277
lectin−24A
lab
Btnd
Alp2
CG6337
CG5804
fabp
zetaTry
lambdaTry
LManII
Cyp4d1
santa−maria
Fer1HCH
thetaTry
MtnB
alphaTry
Mal−A1
Bace
betaTry
LysD
Jon25Biii
5−HT1A
CCHa2
AstA
7B2
Orcokinin
NPF
CngA
AstC
Tk
pros
Sox21a
fru
sbb
hdc
Tet
N
esg
Egfr
E(spl)malpha−BFM
Progenitors
esg−,N+
EE
Immature EC
aEC
mEC−1
mEC−2
pEC−1
pEC−2
EC
CC
cardia
LFC
Unknown−1
Unknown−2
Unknown−3
EE1
EE2
mEC−3
pEC−3
EC−like
Average Expression
Percent Expressed

Figure S3

Figure S3. Transcriptional profile of progenitor-specific IMD inhibition on each epithelial cell type, Related to Figure 3.

A-B: Two dimensional UMAP projection of cell types isolated from female control *esg^ts/+* intestines, and from female *esg^ts/D30A* intestines, color coded by cell type. U = unknown, CC = copper cells, EC = enterocyte, EE = enteroendocrine cell. **C-D:** Heatmap of IEC cluster markers colored by relative gene expression for flies of the indicated genotypes. The size of the dot indicates the proportion of cells in each cluster that expressed the indicated gene.

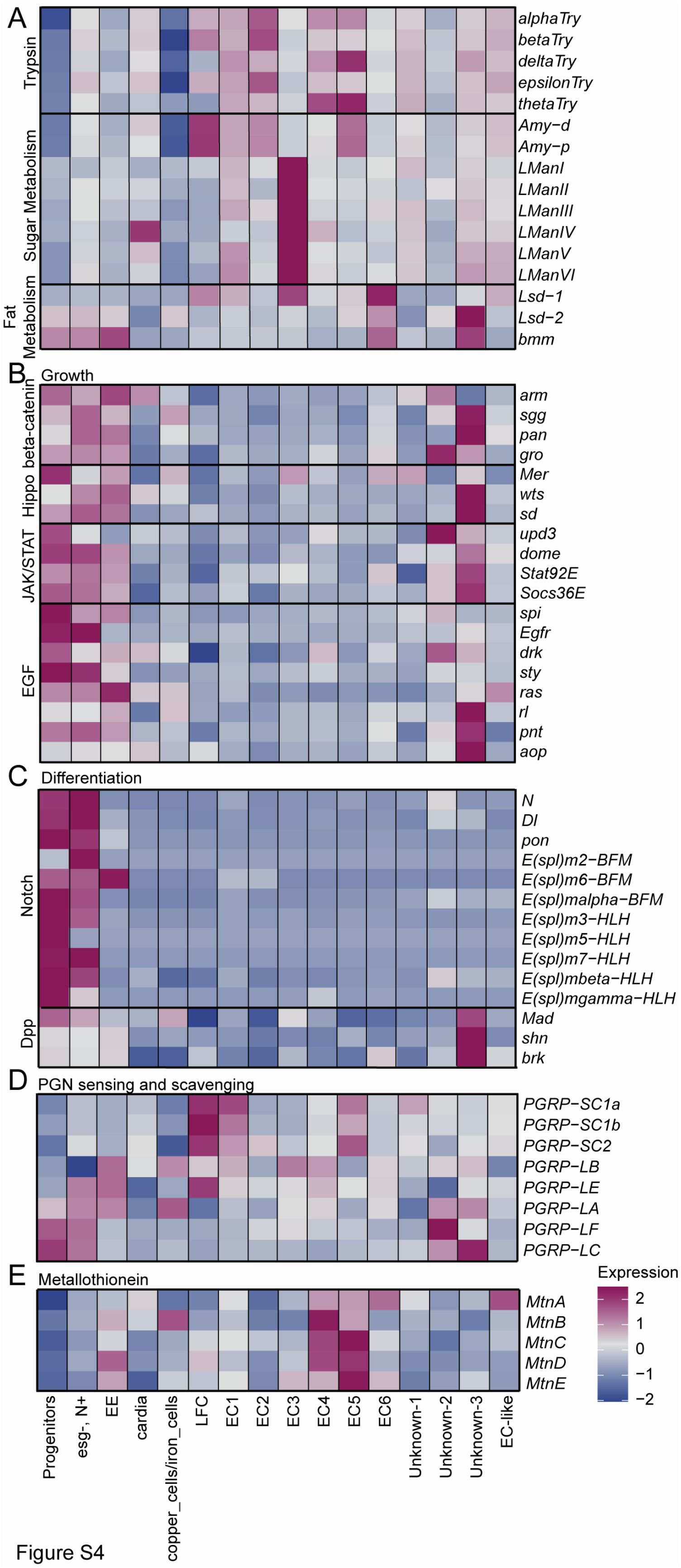
A
Trypsin
Sugar Metabolism
Fat Metabolism
alphaTry
betaTry
deltaTry
epsilonTry
thetaTry
Amy−d
Amy−p
LManI
LManII
LManIII
LManIV
LManV
LManVI
Lsd−1
Lsd−2
bmm
B
Growth
beta-catenin
Hippo
JAK/STAT
EGF
arm
sgg
pan
gro
Mer
wts
sd
upd3
dome
Stat92E
Socs36E
spi
Egfr
drk
sty
ras
rl
pnt
aop
C
Differentiation
Notch
Dpp
N
Dl
pon
E(spl)m2−BFM
E(spl)m6−BFM
E(spl)malpha−BFM
E(spl)m3−HLH
E(spl)m5−HLH
E(spl)m7−HLH
E(spl)mbeta−HLH
E(spl)mgamma−HLH
Mad
shn
brk
D
PGN sensing and scavenging
PGRP−SC1a
PGRP−SC1b
PGRP−SC2
PGRP−LB
PGRP−LE
PGRP−LA
PGRP−LF
PGRP−LC
E
Metallothionein
MtnA
MtnB
MtnC
MtnD
MtnE
Expression
2
1
0
−1
−2
Progenitors
esg-, N+
EE
cardia
copper_cells/iron_cells
LFC
EC1
EC2
EC3
EC4
EC5
EC6
Unknown-1
Unknown-2
Unknown-3
EC-like

Figure S4

Figure S4. Heatmap of metabolism, growth, Differentiation, Peptidoglycan detection, and oxidative stress responses regulators, Related to Figure 3.

A: Heatmap showing relative cluster-average expression of metabolic enzymes (Try = *Trypsin*, Amy = *Amylase*, LMan = Lsysomal alpha-mannosidase, Lsd = Lipid storage droplet, bmm = *brummer*) in each intestinal epithelial cell type of control *esg*^{ts}/+ flies. **B:** Heatmap showing relative cluster-average expression of prominent regulators of intestinal epithelial growth in each intestinal epithelial cell type of control *esg*^{ts}/+ flies. For this analysis, we focused on indicated components of the beta-catenin, Hippo, JAK/STAT, and Epidermal Growth Factor (EGF) pathways.

C: Heatmap showing relative cluster-average expression of prominent regulators of intestinal epithelial differentiation in each intestinal epithelial cell type of control *esg*^{ts}/+ flies. For this analysis, we focused on indicated components of the Notch and Decapentaplegic (Dpp, *Drosophila* ortholog of Bone Morphogenetic Protein) pathways. **D:** Heatmap showing relative cluster-average expression of prominent regulators of Peptidoglycan detection (*PGRP-LE, LC, LA, LF*), and peptidoglycan amidases (*PGRP-SC1a, SC1b, SC2, LB*) in each intestinal epithelial cell type of control *esg*^{ts}/+ flies. For this analysis, we focused on Peptidoglycan Recognition Proteins (PGRP) with detectable expression in the adult intestine. **E:** Heatmap showing relative cluster-average expression of prominent Metallothionein (*Mtn*) regulators of oxidative stress responses in each intestinal epithelial cell type of control *esg*^{ts}/+ flies.

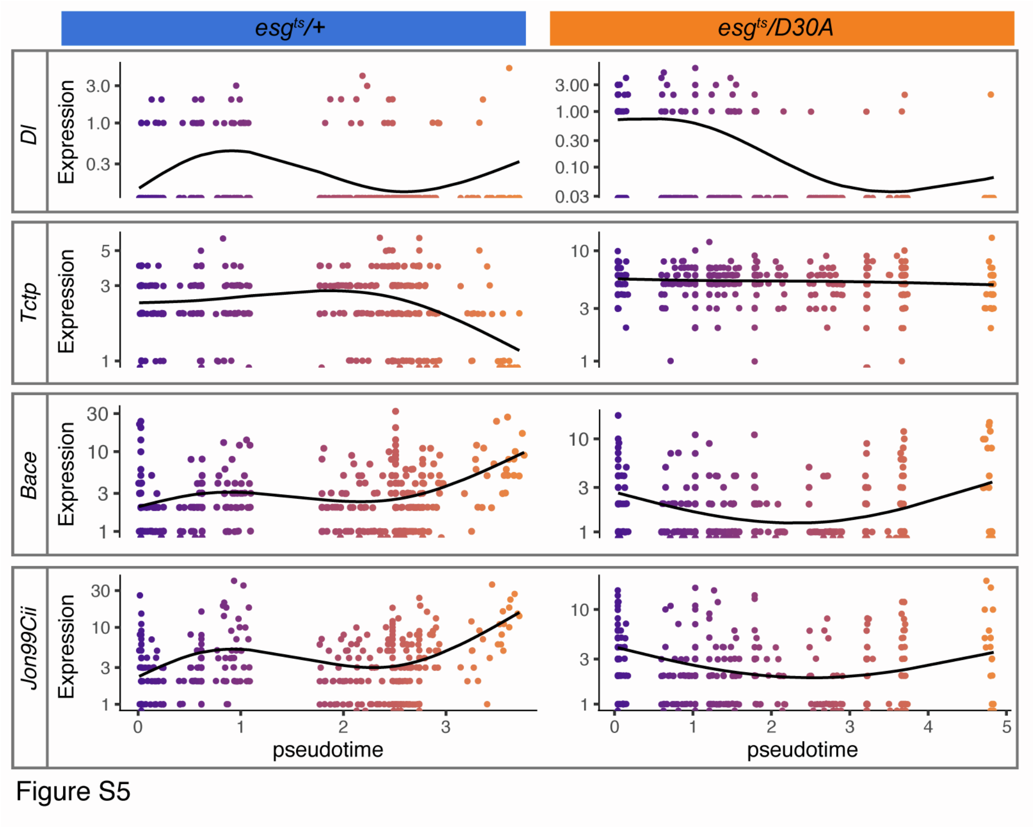

Figure S5. Inhibition of IMD disrupts progenitor cell expression trajectories, Related to Figure 4. Expression of the stem cell marker *Delta (Dl)*, *Translationally-controlled tumor protein (Tctp),* and enterocyte markers *Bace* and *Jon99Cii* along pseudotime in *esg*^{ts}/+ and *esg*^{ts}*/D30A* progenitors. Dark purple marks cells at the beginning of pseudotime while orange marks cells late in pseudotime. Black lines show expression trend.

SUPPLEMENTAL TABLE

Table S1. Inactivation of IMD disrupted expression of 154 genes in progenitors, Related to Figure 1

	p_val	avg_logFC	pct.1	pct.2	p_val_adj	Cluster
**PGRPs**						
PGRP-SC2	0.007338224	0.34923964	0.85	0.827	1	aEC-1
PGRP-LA	0.050117136	-0.06789983	0.065	0.107	1	aEC-1
PGRP-LC	0.277498883	0.104399266	0.097	0.077	1	aEC-1
PGRP-SC1b	0.464238016	-0.06274809	0.053	0.066	1	aEC-1
PGRP-LB	0.162904492	-1.06574588	0.115	0.25	1	aEC-2
PGRP-SC2	0.181166722	0.612109524	0.793	0.75	1	aEC-2
PGRP-SC1b	0.40263009	0.75051183	0.081	0	1	aEC-2
PGRP-SC1a	0.546245626	0.427334596	0.23	0.125	1	aEC-2
PGRP-SC1a	0.00732842	0.26187825	0.222	0.121	1	mEC-1
PGRP-LE	0.01887176	-0.0697811	0.116	0.053	1	mEC-1
PGRP-SC1b	0.17777272	0.09942409	0.091	0.057	1	mEC-1
PGRP-SC2	0.95526117	-0.0728868	0.697	0.621	1	mEC-1
PGRP-SC1b	1.05E-10	0.87502663	0.55	0.156	1.31E-06	mEC-2
PGRP-SC1a	5.05E-10	1.06405117	0.667	0.266	6.33E-06	mEC-2
PGRP-LC	2.14E-05	0.0607342	0.167	0.027	0.26761866	mEC-2
PGRP-LB	2.52E-05	0.22971691	0.483	0.184	0.31613382	mEC-2
PGRP-LF	0.00038527	0.18178081	0.083	0.008	1	mEC-2
PGRP-SD	0.00038527	0.16098043	0.083	0.008	1	mEC-2
PGRP-SC2	0.0023101	0.52052858	0.933	0.98	1	mEC-2
PGRP-LA	0.008040939	-0.1992418	0.034	0.082	1	pEC-1
PGRP-SC1b	0.427890154	0.162931253	0.1	0.085	1	pEC-1
PGRP-LB	5.42E-08	0.67468029	0.36	0.109	0.000679152	pEC-2
PGRP-SC1b	0.452005775	0.069762129	0.08	0.06	1	pEC-2
PGRP-LE	0.581816348	-0.20016583	0.058	0.043	1	pEC-2
PGRP-SC1a	0.745670872	-0.27803406	0.182	0.174	1	pEC-2
PGRP-SC2	0.000989019	0.524798394	0.438	0.256	1	pEC-3
PGRP-LA	0.021084284	-0.39524465	0.177	0.286	1	pEC-3
PGRP-LE	0.106492034	0.173507048	0.285	0.203	1	pEC-3
PGRP-LF	0.169848264	-0.09222145	0.108	0.06	1	pEC-3
PGRP-LB	0.253406709	-0.14482782	0.515	0.391	1	pEC-3
PGRP-LC	0.316279848	-0.05088308	0.077	0.113	1	pEC-3
PGRP-LA	0.054696433	-0.18472686	0.312	0.43	1	pEC-4
PGRP-SC2	0.313823287	-0.10825062	0.729	0.767	1	pEC-4
PGRP-SC1a	0.332124379	0.051890641	0.094	0.058	1	pEC-4
PGRP-LB	0.342758952	-0.09222173	0.938	0.872	1	pEC-4
PGRP-LF	1.89E-07	0.448053148	0.093	0.016	0.002363045	EC-like-1
PGRP-SD	0.000297814	0.156637831	0.053	0.011	1	EC-like-1
PGRP-LB	0.004557101	0.067276621	0.233	0.129	1	EC-like-1
PGRP-LC	0.020479852	0.135834006	0.08	0.037	1	EC-like-1
PGRP-SC1a	0.13461254	-0.14660949	0.26	0.178	1	EC-like-1
PGRP-LF	0.021417831	0.106819364	0.056	0.004	1	EC-like-2
PGRP-LB	0.205049231	-0.71632539	0	0.083	1	EC-like-2
PGRP-SC1b	0.30118776	-0.57823276	0	0.057	1	EC-like-2
PGRP-LE	0.499728118	-0.19528606	0.056	0.026	1	EC-like-2
PGRP-SC2	0.77872758	-0.07259685	0.889	0.605	1	EC-like-2
**AMPs**						
Def	0.0214178	0.1057657	0.056	0.004	1	EC-like-2
Drsl3	0.031586	-0.1106446	0.117	0.043	1	mEC-2

Table S2. Expression of antimicrobial peptides, or peptidoglycan recognition proteins in differentiated enterocytes upon progenitor-specific IMD inhibition, Related to Figure 3.

EXPERIMENTAL PROCEDURES

Ecc15 Oral Infection

For oral infection with *Ecc15*, we incubated an overnight culture of *Ecc15* in LB (Difco^{TM} Luria Broth Base, Miller, 241420) supplemented with NaCl (4.75g Fisher Scientific, BP358-212 per 500mL of LB Broth base) at 29°C with shaking. Flies were starved (10 flies per vial) for 2 h before infection. *Ecc15* was pelleted at 1250g for 10 minutes at 4°C and supernatant decanted. The harvested bacterial pellet was re-suspended in residual LB and an equivalent volume of 5% sucrose in PBS. Flies were transferred into vials that contained a filter paper (Whatman^{TM}, Grade 3, 23mm, 1003-323) soaked with 150ml of the *Ecc15* culture on top of standard corn meal medium. Flies were infected for 16h at 29°C with 12h:12h light:dark cycle. To activate the GeneSwitch (GS) system we added 100µl RU486 (Mifepristone, M8046, Sigma) dissolved in 80% EtOH (5mg/ml) to the surface of standard fool and dried overnight prior to addition of flies. For controls, we added 100µl of 80% EtOH to the surface of standard fool and dried overnight prior to addition of flies. Flies were raised on treated food for 48h prior to infection.

Immunofluorescence

The number of PH-3 or Delta positive cells were analyzed with two-way ANOVA or unpaired Student's t-tests. We used previously described immunofluorescence protocols to visualize posterior midguts (56). In brief, we used anti-phospho-histone H3 (PH3, 1:1000, Millipore (Upstate), 06-570) immunofluorescence to quantify mitoses in the midguts, and anti-Delta (1:100; Developmental Studies Hybridoma Bank (DSHB) C594.9B) immunofluorescence to quantify stem cells in the R4/R5 region of the posterior midguts of virgin female flies that we raised at 29°C. We also used anti-prospero (1:100, DSHB), anti-armadillo (1:100, DSHB) as primary antibodies and Hoechst 33258 (1:500; Molecular Probes) for DNA staining. Secondary antibodies used: goat anti-mouse Alexa Fluor 568 (1:500; Invitrogen), goat anti-rabbit 488 (1:500; Invitrogen). Tissue was mounted in Fluoromount (Sigma-Aldrich F4680) and posterior midguts were visualized with a spinning disk confocal microscope (Quorum WaveFX;

Quorum Technologies Inc.). Images were collected as Z-slices and processed with Fiji software to generate a Z-stacked image.

Isolation of progenitor cell and RNA extraction

Progenitor cells were isolated by fluorescence activated cell sorting (FACS) as previously described by Dutta et al. (2013). Flies were raised at 29°C for 10 days. 100 fly guts per sample were dissected (malphighian tubules, foreguts, hindguts and crops removed) and placed into ice-cold 1XPBS/DEPC-treated water. Guts were dissociated with 1mg/ml of elastase at 27°C with periodic pipetting for 1h. GFP-positive progenitor cells were collected based on GFP fluorescence and size with a BD FACSAriaIII sorter. Cells were pelleted at 1200g for 5 minutes at 4°C and then resuspended in 500µl Trizol. Samples were stored at -80°C until all three biological replicates were collected. RNA was isolated via a standard Trizol-chloroform extraction and the RNA was sent on dry ice to the Lunenfeld-Tanenbaum Research Institute (Toronto, Canada) for library construction and sequencing. The sample quality was evaluated using Agilent Bioanalyzer 2100. TaKaRa SMART-Seq v4 Ultra Low Input RNA Kit for Sequencing was used to prepare full length cDNA. The quality and quantity of the purified cDNA was measure with Bioanalyzer and Qubit 2.0. Libraries were sequenced on the Illumina HiSeq3000 platform.

Preparation of Single Cell Suspension for single cell RNAseq

Single-cell suspension preparation method were followed (Hung et al., 2018) with a few modifications. Flies were raised for 10 days at 29°C. Five guts were dissected at one time and moved to 1% BSA in PBS/DEPC-treated water. Once twenty-seven guts were dissected, we transferred the guts to 200ml 1XPBS/DEPC-treated water on the back side of a glass dissection plate (PYREX, 7220-85) and chopped with scissors. After mechanically fragmenting the tissue, it was transferred to a 1.5 ml tube containing 100ml 1XPBS/DEPC-treated water then enzymatically digested with elastase (final concentration 1mg/ml) at 27°C for 40 min with gentle pipetting every 10 min. The

single cell suspension was pelleted at 300g for 15 min at 4°C and cell pellet resuspended in 200ml 0.04%BSA in 1XPBS/DEPC-treated water. The cell suspension was filtered through a 70μm filter (300g for 1 min at 4°C).

Live cells were collected using OptiPrepTM Density Gradient Medium (SIGMA, D1556-250ML) using the OptiPrepTM Application Sheet C13 protocol. Briefly, a 40% (w/v) iodixanol working solution was prepared with 2 volumes of OptiPrepTM and 1 volume of 0.04 %BSA in 1XPBS/DEPC-treated water. This working solution was used to prepare a 22% (w/v) iodixanol solution in the same buffer. One volume of working solution was carefully mixed with 0.45 volume of cell suspension by gently inversion. The cell suspension/working solution mixture was transferred to a 15ml conical tube then topped up to 6 ml with working solution. The working solution/cell suspension was overlaid with 3 ml of the 22% (w/v) iodixanol and the 22% iodixanol layer was overlaid with 0.5 ml of 0.04 %BSA in 1XPBS/DEPC. Viable cells were separated by density gradient created by centrifuging at 800 g for 20 min at 20°C. Viable cells were harvested from the top interface (~500ul) and then diluted in 2 volumes (1ml) of 0.04 %BSA in 1XPBS/DEPC-treated water. The iodixanol was removed by pelleting live cell suspension at 300 g for 10 min at 4°C. Supernatant was decanted and cells were resuspended in the leftover 0.04 %BSA in 1XPBS/DEPC-treated water. Viability and concentration were measured by 0.4% trypan blue (Gibco, 15250-061) and hemocytometer. Libraries were generated with a 10X Genomics Single-cell Transcriptome Library kit.

Bioinformatics

For purified progenitor RNAseq studies, we obtained approximately 6 million reads per biological replicate. We used FASTQC to evaluate the quality of raw, paired- end reads, and trimmed adaptors and reads of less than 36 base pairs in length from the raw reads using Trimmomatic version 0.36. We used HISAT2 version 2.1.0 to align reads to the *Drosophila* transcriptome- bdgp6, and converted the resulting BAM files to SAM flies using Samtools version 1.8. We counted converted files using Rsubread version 1.24.2 and loaded them into EdgeR. In EdgeR, we filtered genes with counts less than 1 count per million and normalized libraries for size. Normalized libraries were used to call genes that were differentially expressed among treatments. Genes with P-value < 0.05 and FDR < 0.05

were defined as differentially expressed. Principle component analysis was performed on normalized libraries using Factoextra version 1.0.5, and Gene Ontology enRIchment anaLysis and visuaLizAtion tool (GORILLA) was used to determine Gene Ontology (GO) term enrichment. Specifically, differentially expressed genes were compared in a two-list unraked comparison to all genes output from edgeR as a background set, and redundant GO terms were removed.

For single cell analysis, Cell Ranger v3.0 was used to align sequencing reads to the *Drosophila* reference transcriptome (FlyBase, r6.30) and generate feature-barcode matrices. These matrices were analyzed using the Seurat R package (version 3.2.3). Cells possessing <500 UMIs or >2500 UMIs were removed to reduce the number of low- quality cells and doublets. Seurat was then used to normalize expression values and perform integrated data cell clustering at a resolution of 0.5 with 15 principal components. Clusters were identified based on known markers and previous single-cell analysis of the *Drosophila* intestine (https://www.flyrnai.org/scRNA/). For GO term analysis of single cell data, Seurat was used to integrate *esg*^{ts}*/+* and *esg*^{ts}*/D30A* datasets and generate lists of differentially expressed genes for each cluster. Both up- and down-regulated gene lists (p-value cut-off <0.05) were analyzed in GOrilla to determine GO term enrichment. Differentially expressed genes were compared in a two-list unranked comparison to all genes identified in the single- cell dataset. GO terms were then analyzed in REVIGO (REduce and VIsualize Gene Ontology) to remove redundant GO terms. Top enriched GO terms are shown for each cluster, as well as those same GO terms found in other clusters. EE subset analysis was followed at Guo et al. (2019).

For Pseudotime analysis we used Monocle3 (version 0.2.0). Specifically, we converted the existing Seurat data from each genotype separately into a Monocle cell data set of midgut epithelial cells and performed trajectory analysis. We manually assigned the root node of the trajectory to the node at the tip of the Progenitor cluster for each genotype. We then subset the trajectory branch that explains pseudotime within the Progenitor population to perform all subsequent gene level analysis. Here, we manually assessed expression of genes along pseudotime

with known functions in ISC identity, division, and differentiation including genes that were differentially expressed based off our Seurat analysis.

Data availability

Gene expression data have been submitted to the NCBI GEO database (GEO: SuperSeries GSE141897 (GSE171001 and GSE141896)). Single cell gene expression data for *esg*^{ts}*/+* flies (https://singlecell.broadinstitute.org/single_cell/study/SCP1696/single-cell-expression-data-for-d-melanogaster-wild-type-intestines) and for *esg*^{ts}*/D30A* flies (https://singlecell.broadinstitute.org/single_cell/study/SCP1699/single-cell-expression-data-for-d-melanogaster-intestines-with-immune-deficient-progenitor-cells#study-summary) are available for visualization on the Broad Institute Single Cell Portal.
