# Supplementary material for: Immune regulation of intestinal-stem-cell function in Drosophila
Source: Stem Cell Reports. 2022 Mar 17;17(4):741–55. doi: 10.1016/j.stemcr.2022.02.009 (PMC9023782; doi:10.1016/j.stemcr.2022.02.009)
Supplement: Document S2. Article plus supplemental information [file mmc3.pdf]

Stem Cell Reports
Report
OPEN ACCESS

Immune regulation of intestinal-stem-cell function in *Drosophila*

Minjeong Shin,[1] Meghan Ferguson,[1,2] Reegan J. Willms,[1] Lena O. Jones,[1] Kristina Petkau,[1] and Edan Foley[1,2,*]
[1]Department of Medical Microbiology and Immunology Faculty of Medicine and Dentistry University of Alberta Edmonton, Edmonton, AB Canada
[2]Department of Cell Biology Faculty of Medicine and Dentistry University of Alberta Edmonton, Edmonton AB, Canada
*Correspondence: efoley@ualberta.ca
https://doi.org/10.1016/j.stemcr.2022.02.009

SUMMARY

Intestinal progenitor cells integrate signals from their niche, and the gut lumen, to divide and differentiate at a rate that maintains an epithelial barrier to microbial invasion of the host interior. Despite the importance of evolutionarily conserved innate immune defenses to maintain stable host-microbe relationships, we know little about contributions of stem-cell immunity to gut homeostasis. We used *Drosophila* to determine the consequences of intestinal-stem-cell immune activity for epithelial homeostasis. We showed that loss of stem-cell immunity greatly impacted growth and renewal in the adult gut. In particular, we found that inhibition of stem-cell immunity impeded progenitor-cell growth and differentiation, leading to a gradual loss of stem-cell numbers with age and an impaired differentiation of mature enteroendocrine cells. Our results highlight the importance of immune signaling in stem cells for epithelial function in the adult gut.

INTRODUCTION

The intestine is an important contact point between animals and their environments. Intestinal epithelial cells regulate nutrient acquisition, microbiota tolerance, immune education, and pathogen elimination, and disruptions to epithelial homeostasis are linked to inflammatory diseases and cancers. As the epithelium contains a heterogenous population of specialist cell types, it is essential that we understand the mechanisms by which individual lineages regulate intestinal cell proliferation, differentiation, and renewal.

Intestinal epithelial cell (IEC) lineages vary by animal, but data from *Drosophila*, zebrafish, mice, and humans indicate evolutionary conservation of cell-type composition (Brugman, 2016; Buchon et al., 2013a; Lickwar et al., 2017; Miguel-Aliaga et al., 2018; Nguyen et al., 2015; Wallace et al., 2005). Typically, the epithelium is maintained by proliferative, multipotent intestinal stem cells (ISCs) that self-renew and generate all mature epithelial cell types. Most differentiated cells are columnar enterocytes, a cell type specializing in capture and digestion of lumenal nutrients. Secretory-cell-type complexity varies from animal to animal. In flies, the secretory lineage consists solely of hormone-producing enteroendocrine cells. Fish and mammals have mucus-secreting goblet cells in addition to the enteroendocrine population, and mammals also have long-lived, antimicrobial-peptide-producing Paneth cells that neighbor ISCs in basal crypts. ISCs integrate cues from their niche to divide and differentiate at a rate that replenishes dying epithelial cells. Notch, epidermal growth factor (EGF), Wnt, and bone morphogenetic protein (BMP) signal transduction pathways are important regulators of ISC proliferation and differentiation in vertebrates and invertebrates (Spit et al., 2018). Recent studies uncovered roles for immune signaling in ISC survival, growth, and differentiation. For example, vertebrate ISCs express major histocompatibility complex (MHC) class II molecules, and presentation of self-peptides by ISCs appears to be a critical aspect of intestinal invasion and epithelial destruction in graft-versus-host disease (Biton et al., 2018; Fu et al., 2019; Takashima et al., 2019). Likewise, ISCs are enriched for expression of the germline-encoded peptidoglycan receptor NOD2 (Nigro et al., 2014). NOD2 protects ISCs against reactive oxygen species toxicity (Levy et al., 2020), and mutations in NOD2 are associated with Crohn's disease and intestinal tumorigenesis (Couturier-Maillard et al., 2013). Despite established requirements for immune-signaling pathways in the maintenance of intestinal health, it is unclear if innate defenses act specifically in progenitors to regulate epithelial homeostasis. We consider this an important knowledge gap given the central role of intestinal progenitors in building and maintaining the entire epithelium.

Drosophila melanogaster are widely used to characterize intestinal immunity and homeostasis. The adult fly intestine is a pseudostratified epithelium that is maintained by multipotent ISCs (Micchelli and Perrimon, 2006; Ohlstein and Spradling, 2006). The majority of ISC divisions are asymmetric, producing a new ISC, and are transient cell types that generate terminally differentiated epithelial cells. In most cases, ISC divisions generate a post-mitotic enteroblast that differentiates as an enterocyte in response to Notch signals (Bardin et al., 2010; Guo and Ohlstein, 2015; Ohlstein and Spradling, 2007). Collectively, ISCs and enteroblasts are classified as the intestinal progenitor compartment in flies. In the absence of cues from Notch, ISCs transition through a pre-enteroendocrine state to generate mature enteroendocrine cells that can be

This is an open access article under the CC BY license (http://creativecommons.org/licenses/by/4.0/).

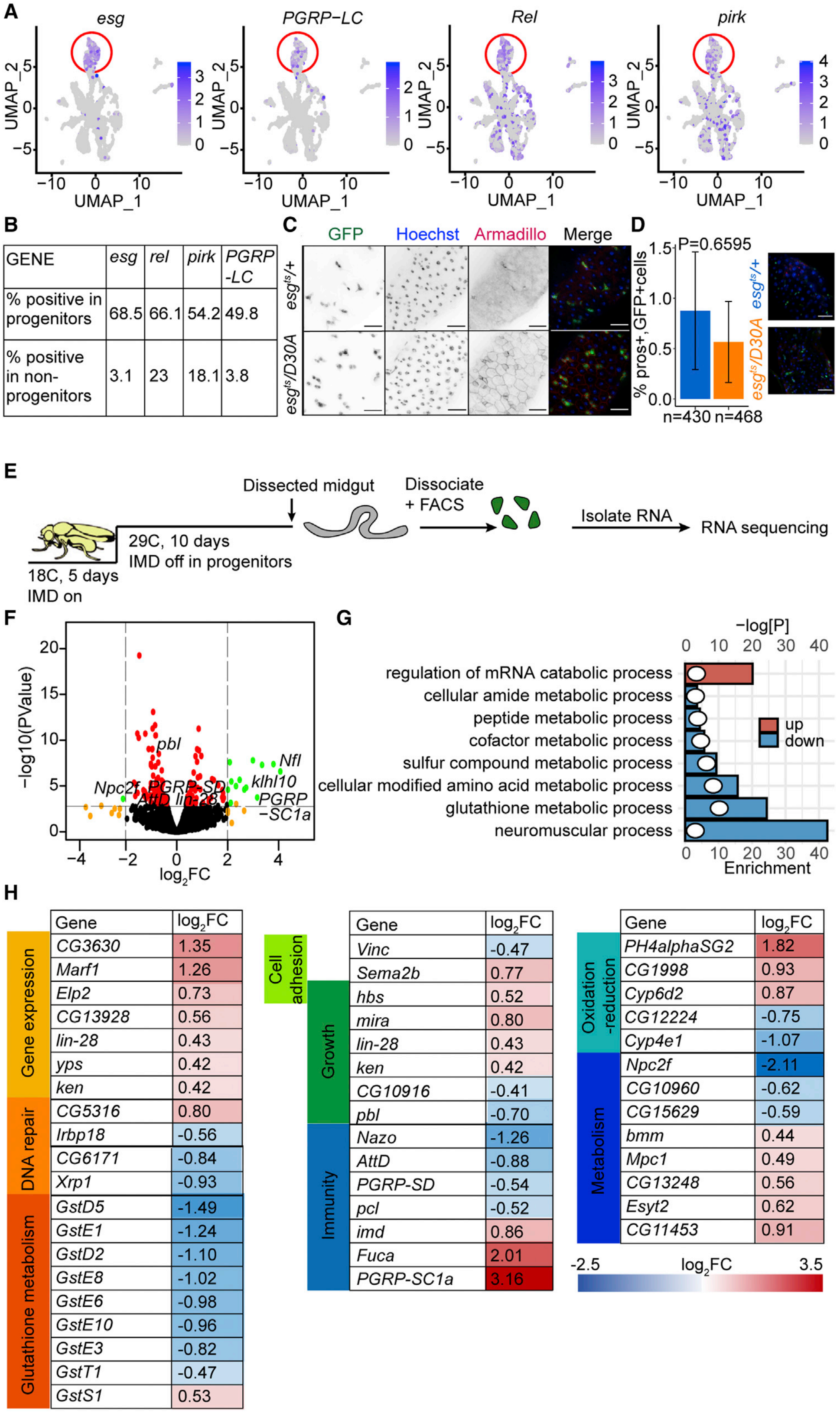

(legend on next page)

sub-classified into functional groups based on intestinal localization and hormone expression patterns (Biteau and Jasper, 2014; Guo and Ohlstein, 2015; Zeng and Hou, 2015). In the fly gut, bacterial diaminopimelic acid-type peptidoglycan (PGN) activates immune responses via the immune deficiency (IMD) pathway, a germline-encoded defense with similarities to vertebrate tumor necrosis factor receptor signaling (Buchon et al., 2009b, 2013a; Myllymäki et al., 2014). Detection of extracellular PGN by the PGN recognition protein LC (PGRP-LC) receptor, or intracellular PGN by the related PGRP-LE receptor, converges on a signaling complex that includes the Imd protein, the adaptor protein Fas-associated death domain (FADD), and the Caspase-8 homolog, Dredd. Dredd removes thirty N-terminal amino acids from Imd, initiating molecular events that activate c-Jun N-terminal kinase, and the p100/105 nuclear factor κB (NF-κB) ortholog Relish (Rel) (Leulier et al., 2000; Stoven et al., 2003; Stöven et al., 2000). Thus, Dredd-mediated processing of Imd is essential for IMD pathway activation, and expression of a non-cleavable Imd variant (ImdD30A) blocks host responses to PGN in cell culture and *in vivo* (Kim et al., 2014; Paquette et al., 2010). In the fly intestine, Rel and c-Jun N-terminal kinase initiate transcriptional responses that include regionalized expression of antimicrobial peptides, regulators of metabolism, and genes associated with growth and differentiation (Broderick et al., 2014; Buchon et al., 2009b, 2009a; Dutta et al., 2015; Hung et al., 2020). Earlier work uncovered significant differences between the responses of mature epithelial cell types to IMD activation. For example, infection-dependent activation of IMD in enterocytes results in antimicrobial peptide expression and extrusion of damaged cells (Buchon et al., 2009b; Dutta et al., 2015; Zhai et al., 2018). In contrast, activation of IMD in enteroendocrine cells by bacterial lactate modifies lipid metabolism in neighboring enterocytes (Kamareddine et al., 2018). Notably, genomic studies demonstrated expression of IMD pathway components in intestinal progenitor cells (Dutta et al., 2015; Hung et al., 2020). However, the contributions of progenitor-specific IMD activity to intestinal homeostasis are unexplored.

We took advantage of the genetic accessibility of flies to ask if progenitor-specific IMD affects intestinal homeostasis in *Drosophila*. Specifically, we used genomic and physiological assays to determine the consequences of blocking IMD in intestinal progenitors. We found that inhibition of progenitor-cell IMD had significant effects on ISC proliferation, progenitor compartment composition, and generation of mature enteroendocrine cells. As germline-encoded immune responses are known modifiers of vertebrate intestinal epithelial growth, we believe our findings are of general relevance to understanding how host immune responses control stem-cell function in the intestine.

RESULTS

IMD regulates the intestinal progenitor-cell transcriptome

In a single-cell RNA sequencing profile of adult female *Drosophila* intestines, we identified 620 cells that expressed the progenitor-cell markers *esg*, *Dl*, and *N* (Figure S1). Within the progenitors, we also observed enriched expression of key IMD pathway components, including the PGN sensor *pgrp-lc*, the NF-kB transcription factor *relish*, and the IMD pathway target *pirk* (Figures 1A and 1B). Our data match earlier reports of IMD pathway gene expression in progenitors (Dutta et al., 2015; Hung et al., 2020) and raise the possibility that immune signals contribute to gut-progenitor-cell function.

To test IMD activity in progenitors, we used the *esgGAL4*, *GAL80^{ts}*, *UASGFP* (*esg^{ts}*) fly line to express a dominant inhibitory IMD protein (ImdD30A) in *Drosophila*

Figure 1. IMD regulates the intestinal progenitor-cell transcriptome

(A) Uniform manifold approximation and projection (UMAP) plot of intestinal epithelial cells isolated from 10-day-old *esg^{ts}* flies showing expression of the progenitor marker *esg* and the IMD pathway components *PGRP-LC*, *Rel*, and *pirk*. Progenitors are circled in red.

(B) Quantification of the percentage of progenitors and non-progenitors that express the indicated genes.

(C) Visualization of GFP, DNA (Hoechst), and the beta-catenin ortholog in intestines of 10-day-old *esg^{ts}*/+ and *esg^{ts}/UAS-imdD30A* (*esg^{ts}/D30A*) flies. Scale bars represent 25 μm.

(D) Quantification of the percentage of GFP-positive cells that express the enteroendocrine cell marker *prospero* in *esg^{ts}*/+ (n = 20) and *esg^{ts}/D30A* (n = 22) flies. *esg^{ts}*/+ (n = 430) and *esg^{ts}/D30A* (n = 468) n = number of GFP+ cells. Representative images are shown with DNA in blue, *esg*+ cells in green, and *prospero*-positive enteroendocrine cells in red. Significance measured using Student's t test. Scale bars represent 25 μm.

(E) Schematic representation of an experimental strategy to quantify gene expression in progenitor cells purified from *esg^{ts}*/+ and *esg^{ts}/D30A* flies. Each experiment was performed in triplicate.

(F) Volcano plot showing relative changes (x axis) and significance (y axis) in gene expression of purified progenitors from *esg^{ts}/D30A* flies compared with age-matched *esg^{ts}*/+ controls. Genes are color-coded to indicate significance and relative gene expression changes.

(G) Gene Ontology analysis of processes significantly affected by inhibition of IMD in progenitors. Column size indicates the degree of enrichment for each term, and dots indicate the log-transformed significance of the respective enrichment.

(H) Representative sample of genes with affected expression upon inhibition of IMD in progenitors. See also Figures S1 and S2.

progenitors (esg^{ts}/*D30A*) for 10 days. Blocking IMD in progenitors did not stop infection-mediated expression of IMD-responsive antimicrobial peptides in enterocytes, demonstrating that inhibition of progenitor-cell IMD does not affect IMD activity in differentiated progeny (Figure S2). We then asked if blocking IMD had direct effects on the progenitor population. Both esg^{ts}/*D30A* and control esg^{ts}/+ intestines had similar distributions of small, GFP-positive cells (Figure 1C) that rarely expressed the enteroendocrine cell marker *prospero* (Figure 1D), confirming that GFP exclusively marked progenitors in both lines. To measure effects of IMD on progenitors, we performed RNA sequencing (RNA-seq) analysis of gene expression in fluorescence-activated cell sorting (FACS)-purified GFP-positive cells from esg^{ts}/*D30A* and esg^{ts}/+ flies (Figure 1E). We found that inactivation of IMD disrupted expression of 154 genes in progenitors (Figure 1F; Table S1), including key IMD pathway regulators (*pgrp-sd*, *pgrpsc1a*), glutathione metabolism genes required for detoxification of xenobiotic substances, and 24 genes known to respond to the commensal microbiome (Broderick et al., 2014) (Figures 1F–1H). Notably, the impacts of IMD inhibition extended beyond conventional antimicrobial responses and included diminished expression of genes associated with stem-cell growth and adhesion to the niche, such as the growth regulator *Xrp1*, the asymmetric cell division regulator *miranda* (*mira*), and the effector of extracellular matrix adhesion *Vinculin* (*Vinc*; Figures 1F–1H), suggesting potential growth-regulatory roles for IMD in progenitors.

IMD modifies ISC division

As IMD inhibition affected the progenitor transcriptome, we asked if IMD also affects progenitor homeostasis. Specifically, we measured ISC mitoses by quantifying phosphohistone H3, the percentage of midgut epithelial cells that expressed the progenitor marker *esg*, and the percentage of progenitors that expressed the ISC marker *Delta* (*Dl*) in 5- and 30-day-old esg^{ts}/*D30A* and esg^{ts}/+ intestines. In young esg^{ts}/+ intestines, we observed few ISC divisions (Figure 2A), approximately 20% *esg*+ cells in the posterior midgut (Figure 2B), and 40% Dl+ ISCs per progenitor (Figure 2C). Consistent with reports of age-related decline in gut function, we detected significantly increased numbers of ISC divisions, *esg*+ cells, and Dl+ cells in aged esg^{ts}/+ intestines relative to their 5-day-old counterparts (Figures 2A–2C). In contrast, we did not detect age-dependent increases in mitoses, progenitor numbers, or ISC numbers in 30-day-old esg^{ts}/*D30A* intestines (Figures 2A–2C). Instead, 30-day-old esg^{ts}/*D30A* intestines were characterized by significantly fewer mitoses, Dl+ ISCs, and progenitors than 30-day-old esg^{ts}/+ control flies. Importantly, these results are not an artifact of *imdD30A* expression, as progenitor-specific, RNAi-mediated depletion of the IMD pathway adaptor FADD caused a significant decline in the amounts of Dl-positive stem cells among intestinal progenitors of 30-day-old flies (Figure 2D). Furthermore, progenitor-specific inactivation of *relish* significantly impaired generation of mitotic clones in the posterior midgut (Figure 2E), confirming that genetic inhibition of IMD blocks intestinal epithelial proliferation. ISC-specific inactivation of IMD was sufficient to block proliferation (Figure 2F), whereas EB-specific inhibition of IMD had no effect (Figure 2G), suggesting a cell-autonomous role for IMD in controlling ISC division. Finally, we discovered that inhibition of the PGN sensors *PGRP-LC* or *PGRP-LE* (Figures 2H–2I) was sufficient to inhibit progenitor-cell proliferation. Collectively, our data indicate that inactivation of IMD in progenitors significantly impairs age-dependent accumulation of mitotically active progenitors in the adult midgut. We consider these findings particularly interesting as increased epithelial immune responses are a hallmark of the aging intestine (Broderick et al., 2014; Buchon et al., 2009a; Guo et al., 2014; Ren et al., 2007).

Single-cell analysis uncovers impacts of progenitor-cell IMD on the intestinal epithelium

Similar to vertebrates, the *Drosophila* intestine is a highly heterogenous tissue. Multipotent stem cells generate distinct epithelial lineages that control nutrient acquisition, hormone production, and responses to intestinal microbes in a regionally specialized fashion. Thus, although our data implicate IMD in progenitor-cell division, we do not yet understand the consequences of blocking IMD in progenitors for the entire intestine. To determine the effects of progenitor-specific IMD inhibition on all epithelial cell types, we resolved the transcriptomes of 10-day-old esg^{ts}/+ and esg^{ts}/*D30A* intestines at the single-cell level (Figures S3A and S3B). After excluding dead cells and doublets, we prepared RNA sequencing profiles of 3,675 cells from esg^{ts}/+ intestines and 3,654 cells from esg^{ts}/*D30A* intestines. Using unsupervised graph-based clustering of data from esg^{ts}/+ intestines, we identified all cell types previously described in the adult gut, including progenitors that expressed growth and differentiation regulators, enteroendocrine cells that produced peptide hormones, and enterocytes dedicated to digestion (Figure S3). A more detailed examination of single-cell transcriptomes from esg^{ts}/+ intestines uncovered clear signs of specialization among the individual cell types. Specifically, we discovered regionalized and cell-type-specific expression patterns for regulators of metabolism, growth, differentiation, PGN sensing and scavenging, and oxidative stress responses (Figure S4). Thus, our profile of esg^{ts}/+ guts accurately recapitulated known features of spatial and functional specialization within the fly gut (Dutta et al., 2015; Hung et al., 2020), providing a reliable control for analysis of intestines with impaired progenitor-cell IMD.

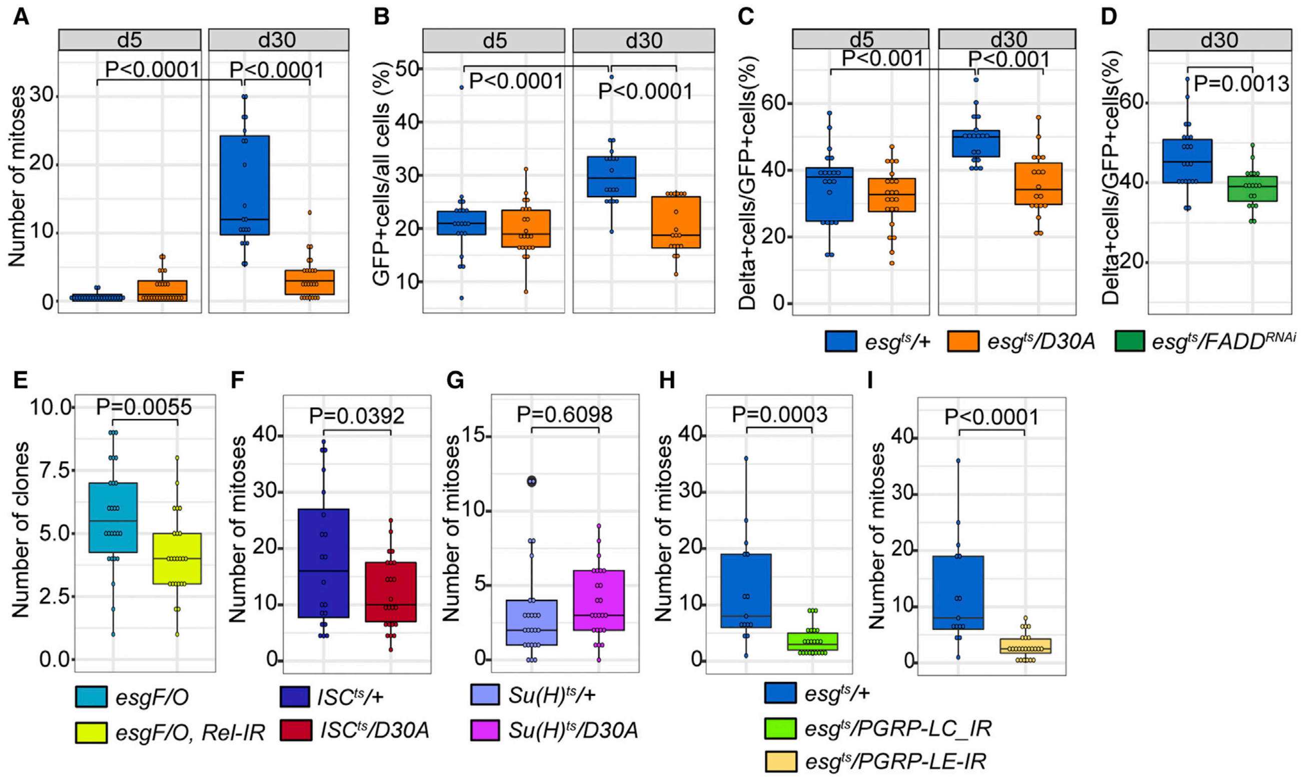

Figure 2. Progenitor-cell IMD activity modifies stem-cell proliferation

(A) Quantification of mitoses per gut in intestines from *esg*^{ts}/+ and *esg*^{ts}/*D30A* flies of the indicated ages.

(B) Percentage of intestinal epithelial cells that express the progenitor marker *esg* in *esg*^{ts}/+ and *esg*^{ts}/*D30A* flies.

(C) Percentage of progenitors that express the stem-cell marker Delta in *esg*^{ts}/+ and *esg*^{ts}/*D30A* flies.

For (A)–(C), n = 20 at day 5 and 18 at day 30 in *esg*^{ts}/+ flies and n = 22 at day 5 and 18 at day 30 in *esg*^{ts}/*D30A* flies.

(D) Percentage of progenitors that express the stem-cell marker Delta in *esg*^{ts}/+ (n = 21) and *esg*^{ts}/*FADD*^{RNAi} (n = 18) flies.

(E) Quantification of GFP-marked mitotic clones in the posterior midgut of *esg*$^{F/O}$ (n = 26) and *esg*$^{F/O}$, *rel-IR* (n = 24) flies 9 days after marking of mitotic clones.

(F) Quantification of mitoses per gut in *ISC*^{ts}/+ (n = 21) and *ISC*^{ts}/*D30A* (n = 24) 27-day-old flies.

(G) Quantification of mitoses per gut in 27-day-old *Su(H)*^{ts}/+ (n = 25) and *Su(H)*^{ts}/*D30A* (n = 24) flies.

(H and I) Quantification of mitoses per gut in 27-day-old *esg*^{ts}/+ (n = 15), *esg*^{ts}/*PGRP-LC-IR* (n=22) (H) and *esg*^{ts}/*PGRP-LE-IR* (n = 24) (I) flies.

Statistical significance for (A)–(C) was calculated using an ANOVA followed by pairwise Tukey comparisons, and significance for (D)– (I) was calculated using a Student's t test.

To determine if blocking IMD in progenitors affects mature epithelial cells, we used the integrated data analysis workflow in Seurat to identify cell-type-specific differences in gene-expression patterns between *esg*^{ts}/+ and *esg*^{ts}/*D30A* intestines. Unsupervised clustering of the integrated data resolved progenitors, enterocytes, and enteroendocrine cells, as well as cardia, copper cells, an enterocyte-like cluster, a cluster of immature enterocytes, and three lineages of unknown function (Figure 3B). Comparisons between the two genotypes suggested mild effects of blocking IMD in progenitors on the generation of mature IECs. For example, we noted fewer enterocyte (EC)-like cells and considerably more cardia in intestines from *esg*^{ts}/*D30A* than in *esg*^{ts}/+ flies (Figure 3B). Furthermore, blocking IMD in progenitors affected the expression of genes associated with critical regulatory functions in the gut. For example, intestines from *esg*^{ts}/*D30A* flies were characterized by shifts in RNA processing and translation in ECs, diminished precursor metabolite generation in enteroendocrine cells, and increased expression of genes involved in autophagy, cell polarity, and adhesion in progenitors (Figure 3C). Notably, blocking IMD in progenitors did not affect expression of antimicrobial peptides, or PGN recognition proteins, in differentiated ECs (Table S2), further arguing that expression of *imdD30A* in progenitor cells does not inhibit immune activity in progeny. Instead, we observed substantial effects of inhibiting IMD on expression of genes with essential roles in progenitor-cell division and polarity (Figure 3D),

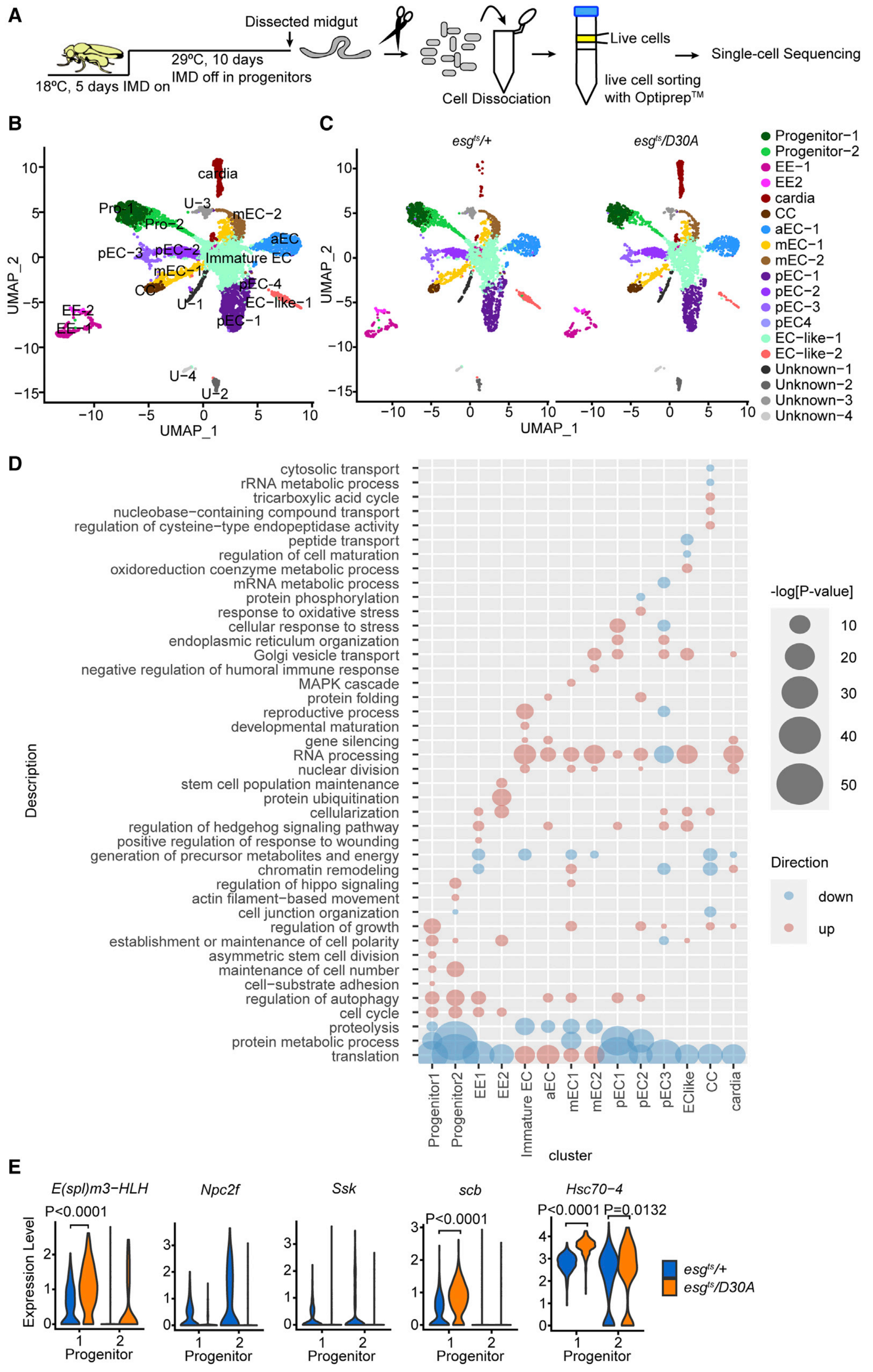

(legend on next page)

including the Notch signaling modifier *Npc2f*, the Notch pathway target *E*(*spl*)*m3-HLH*, and *Snakeskin* (*Ssk*) a key regulator of intestinal-stem-cell activity (Figure 3E). Thus, and consistent with data presented in Figures 1 and 2, our results indicate that inhibition of IMD in progenitors has significant effects on progenitor-cell homeostasis.

As we believe our gene expression data are likely of value to the community outside the scope of the current study, we have deposited both sets on the Broad Institute Single Cell Portal (see experimental procedures for further details).

IMD affects developmental trajectories within the progenitor compartment

We were intrigued by our observation that blocking IMD in progenitors significantly affected expression of genes required for progenitor-niche interactions and progenitor differentiation. Therefore, we used Monocle to prepare pseudotime developmental trajectories for esg^{ts}/+ and esg^{ts}/*D30A* intestines. Analysis of the respective datasets successfully re-created developmental transitions from multipotent progenitors to differentiated lineages in both lines (Figures 4A and 4D). To directly examine effects of IMD on progenitors, we created subsets of the progenitor population for each genotype and analyzed gene expression in pseudotime for the respective subsets. Examination of the progenitor population from both genotypes revealed gene-expression patterns characteristic of developmental transitions along a pseudotime trajectory (Figures 4B and 4E). For example, both progenitor populations were characterized by expression of the ISC marker *Dl* in early stages of pseudotime (Figures 4C and 4F). However, IMD inhibition resulted in premature and prolonged pseudotime expression of the Notch targets *E(spl)m3-HLH* (Figures 4G and 4H) and *E(spl)malpha-BFM* (Figures 4I and 4J), the EGF regulator *sprouty* (*sty*; Figures 4K and 4L), the EC fate regulator *klumpfuss* (*klu*; Figures 4M and 4N), and numerous markers of EC maturation (Figure S5), indicating effects of progenitor-cell IMD on EC differentiation. To test if blocking IMD in progenitors impacts the transition from ISC to enteroblast, we monitored expression of fluorescent markers in *esgGAL4*, *UAS-CFP*, and *Su(H)-GFP; GAL80*^{ts} flies that expressed ImdD30A. In these flies, ISCs are visible as CFP-positive cells (pseudocolored as yellow), and enteroblasts are visible as CFP and GFP double-positive cells (pseudocolored as magenta). Consistent with putative interactions between IMD and Notch, we found that blocking IMD significantly increased the percentage of progenitors that expressed the enteroblast marker *Su*(*H*)*-GFP* (Figures 4O and 4P). Thus, in agreement with the loss of ISCs noted in esg^{ts}/*D30A* intestines (Figure 2), our data argue that IMD activity influences progenitor-cell composition in the fly intestinal epithelium.

Progenitor IMD affects generation of mature enteroendocrine cells

IMD inhibition impaired ISC proliferation, diminished ISC numbers, and impacted cell-type composition within the progenitor compartment, suggesting possible effects of progenitor-cell IMD on the development of mature epithelial cells. To determine if inhibition of IMD in progenitors affects epithelial differentiation, we monitored the *prospero*-positive enteroendocrine (EE) cell population in esg^{ts}/+ and esg^{ts}/*D30A* intestines. We focused on EE cells, as fly EE cells have been characterized to a single-cell resolution (Guo et al., 2019; Hung et al., 2020), permitting detailed comparisons between esg^{ts}/+ and esg^{ts}/*D30A* intestines. *Drosophila* EE cells can be divided into subsets with distinct peptide-hormone expression profiles that are stable during homeostasis or after recovery from infection (Beehler-Evans and Micchelli, 2015). Therefore, we tested if inhibition of IMD in progenitors affected the representation of EE subsets in the intestine. Using unsupervised clustering, we found that EE cells clustered into five subsets in both genotypes (Figures 5A and 5B). In both genotypes, each EE subset had a signature hormone-expression pattern (Figures 5C and 5D). In some cases, subset-restricted expression patterns were conserved between esg^{ts}/*D30A* and esg^{ts}/+ EE

Figure 3. Inactivating IMD in progenitors affects transcriptional activity in all intestinal epithelial cell types

(A) Schematic representation of an experimental strategy for single-cell transcriptomic analysis of purified intestinal epithelial cells from esg^{ts}/+ and esg^{ts}/*D30A* flies.

(B) UMAP plot visualizing cell types in integrated data from esg^{ts}/+ and esg^{ts}/*D30A* flies based on the expression of marker genes. Cells are color-coded by cell type.

(C) The same data from (B), split into the labeled genotypes. EE, enteroendocrine cells; CC, copper cells; EC, enterocytes subdivided according to anterior-posterior distribution along the intestine (a, anterior; m, middle; p, posterior).

(D) Gene Ontology term analysis of cell-type-specific processes significantly affected by progenitor-restricted inhibition of IMD. Bubble size indicates the log-transformed significance of the respective enrichments. Pink bubbles indicate enhanced terms, and blue bubbles indicate underrepresented terms.

(E) Representative violin plots of expression levels for the indicated genes in progenitors of esg^{ts}/+ and esg^{ts}/*D30A* flies. p values indicate significantly different expression levels. For *Npc2f* and *Snakeskin* (*Ssk*), no expression was observed in progenitors of esg^{ts}/*D30A* flies. See also Figures S3 and S4.

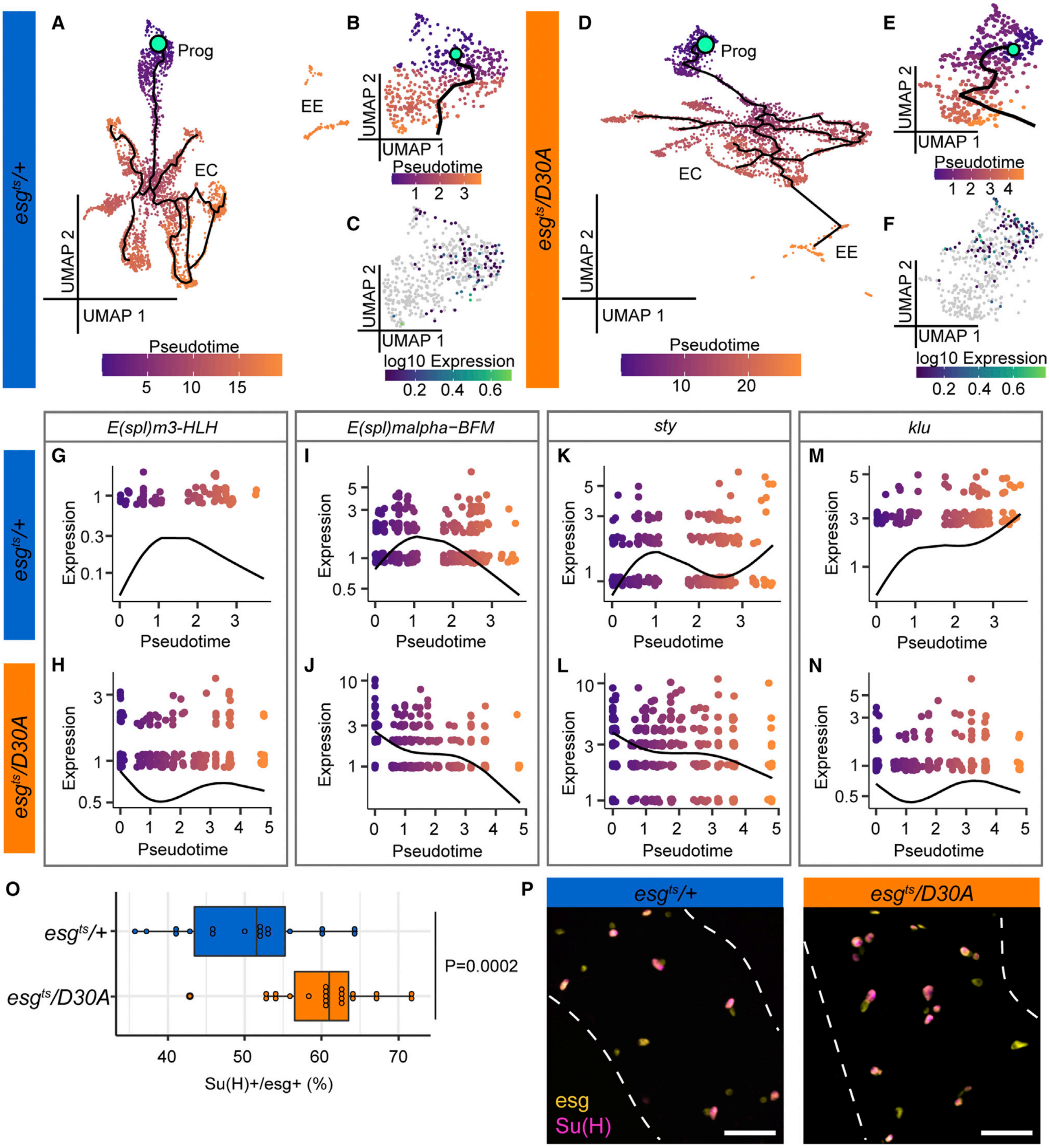

Figure 4. Inhibition of IMD affects developmental trajectories within the progenitor compartment

Single-cell datasets from Seurat for each individual genotype were loaded into Monocle3, and pseudotime analysis was performed on midgut epithelial cells.

(A and D) *esg*^{ts}/+ midguts with wild-type progenitors (A) and *esg*^{ts}*/D30A* midguts with IMD-deficient progenitors (D). Mint green circles denote the root node and beginning of the intestinal trajectories. Dark purple marks cells at the beginning of pseudotime, while orange marks cells late in pseudotime. Black lines show trajectories. Prog, progenitors; EC, enterocytes; EE, enteroendocrine cells.

(B and E) Pseudotime within progenitor subsets of (A) and (D), respectively.

(legend continued on next page)

cells. For example, matching an earlier characterization of EE subsets (Beehler-Evans and Micchelli, 2015), we found that cells from *esg*^{ts}/+ subset zero, and from *esg*^{ts}/*D30A* subset two, expressed *Tk* and *Dh31*. Likewise, *esg*^{ts}/+ subset three cells and *esg*^{ts}/*D30A* subset one cells were characterized by enhanced expression of *NPF* and by partial expression of *Gbp5* and *CCAP*. In contrast, we did not detect a counterpart of *esg*^{ts}/*D30A* subset zero cells in *esg*^{ts}/+ controls, and we saw minimal conservation of *esg*^{ts}/+ subset zero gene expression patterns in *esg*^{ts}/*D30A* EE cells, suggesting functional differences between EE cells in *esg*^{ts}/*D30A* flies compared with *esg*^{ts}/+ flies. When we classified EE cells based on the number of peptides they expressed, we noted further differences between *esg*^{ts}/+ and *esg*^{ts}/*D30A* intestines. In particular, fewer EE cells expressed zero or one peptide in *esg*^{ts}/*D30A* guts than in *esg*^{ts}/+ guts, and a greater proportion expressed two or more peptides (Figure 5E). Likewise, for thirteen of fourteen peptides examined, a greater percentage of *esg*^{ts}/*D30A* EE cells expressed the respective peptide than *esg*^{ts}/+ controls (Figure 5F), indicating enhanced peptide expression in *esg*^{ts}/*D30A* flies. To directly test the effects of blocking IMD in progenitors on peptide-expression levels, we performed an RNA-seq analysis of dissected midguts from *esg*^{ts}/*D30A* and *esg*^{ts}/+ flies. With the exceptions of *ITP* and *Gpb5*, we found that blocking IMD in progenitors resulted in increased expression of the remaining twelve peptides (Figure 5G), confirming a link between IMD inhibition and peptide-hormone expression. Finally, we quantified EE numbers in posterior midguts of *esg*^{ts}/+ and *esg*^{ts}/*D30A* flies. We found that inhibition of IMD in progenitors decreased the proportion of mature EE cells by roughly 20% relative to *esg*^{ts}/+ controls (Figure 5H). Combined, our results establish that inhibition of progenitor-cell IMD disrupts peptide-hormone expression patterns in mature EE cells, decreases the amount of total EE cells, and increases the expression of most peptide hormones, confirming a link between progenitor-cell IMD activity and EE-cell development.

DISCUSSION

Notch, BMP, and WNT pathways regulate intestinal progenitor-cell growth and differentiation in vertebrates and invertebrates (Barker, 2014; Casali and Batlle, 2009; Miguel-Aliaga et al., 2018; Sancho et al., 2015; Vooijs et al., 2011; Xu et al., 2011). In contrast, it is less clear what effects progenitor-specific activation of germline-encoded immune responses has on epithelial homeostasis. Several studies indicated survival and growth-regulatory effects of host immunity on intestinal progenitors. For example, the PGN receptor NOD2 is enriched in ISCs of mice (Nigro et al., 2014) and protects ISCs from irradiation-induced cytotoxicity (Levy et al., 2020), while mutations in NOD2 are linked to Crohn's disease (Hugot et al., 2001; Ogura et al., 2001). Likewise, TLR4 is expressed to higher levels in intestinal crypts (Price et al., 2018), where its activation promotes apoptosis and inhibits proliferation (Naito et al., 2017; Neal et al., 2012). In contrast, epithelium-wide activation of TLR4 promotes epithelial repair by activating EGF and JAK/STAT pathways in mice challenged with dextran sulfate sodium (Fukata et al., 2006; Hsu et al., 2010). These studies support roles for immune pathways in proliferative responses to extrinsic challenges. However, we do not know if progenitor-specific immune activity impacts homeostatic growth and differentiation. We consider this an important question, as intestines contain dense microbial communities that promote growth and influence cell-fate choices in intestinal epithelia (Ferguson and Foley, 2021).

We measured growth and differentiation in adult *Drosophila* midguts that we engineered to lack IMD activity in progenitors. The IMD pathway is highly similar to mammalian tumor necrosis factor receptor signaling, and IMD exerts broad regulatory effects on intestinal transcription (Broderick et al., 2014). In flies, IMD has context-dependent effects on ISC proliferation. IMD-pathway mutants have elevated rates of mitoses that are driven by the microbiome (Buchon et al., 2009b; Guo et al., 2014; Paredes et al., 2011), but IMD is not required for the proliferative burst observed after challenges with *Ecc15* (Zhai et al., 2018). In contrast, infection with *Vibrio cholerae* blocks ISC proliferation in an IMD-dependent manner (Fast et al., 2020; Wang et al., 2013), whereas *Herpetomonas muscarum* induces IMD-dependent proliferation (Wang et al., 2019). In the absence of infection, persistent activation of IMD in progenitors increases ISC division frequency and skews differentiation towards elevated numbers of EE cells (Petkau et al., 2017). Similarly, overexpression of PGRP-LC in ECs induces Rel-dependent proliferation (Zhai et al.,

(C and F) *Delta* (*Dl*) expression patterns within *esg*^{ts}/+ progenitors (C) and *esg*^{ts}/*D30A* progenitors (F). Gray dots are cells with no detectable expression.

(G–N) Expression of Notch target genes *E(spl)m3-HLH*, *E(spl)malpha-BFM,* the EGF inhibitor *sprouty* (*sty*), and the EC fate regulator *klumpfuss* (*klu*) over pseudotime within progenitor subsets of the indicated genotypes.

(O) Percent of *esg*+ progenitors that are positive for the enteroblast marker *Su(H)*+ in *esg*^{ts}*, UAS-CFP, Su(H)-GFP*/+ (n = 18) and *esg*^{ts}*, UAS-CFP, Su(H)-GFP /D30A* (n = 22) posterior midguts 14 days after transgene expression. Significance found using Student's t test.

(P) Representative images of intestines used to gather data for (O). Scale bars represent 25 μm. See also Figure S5.

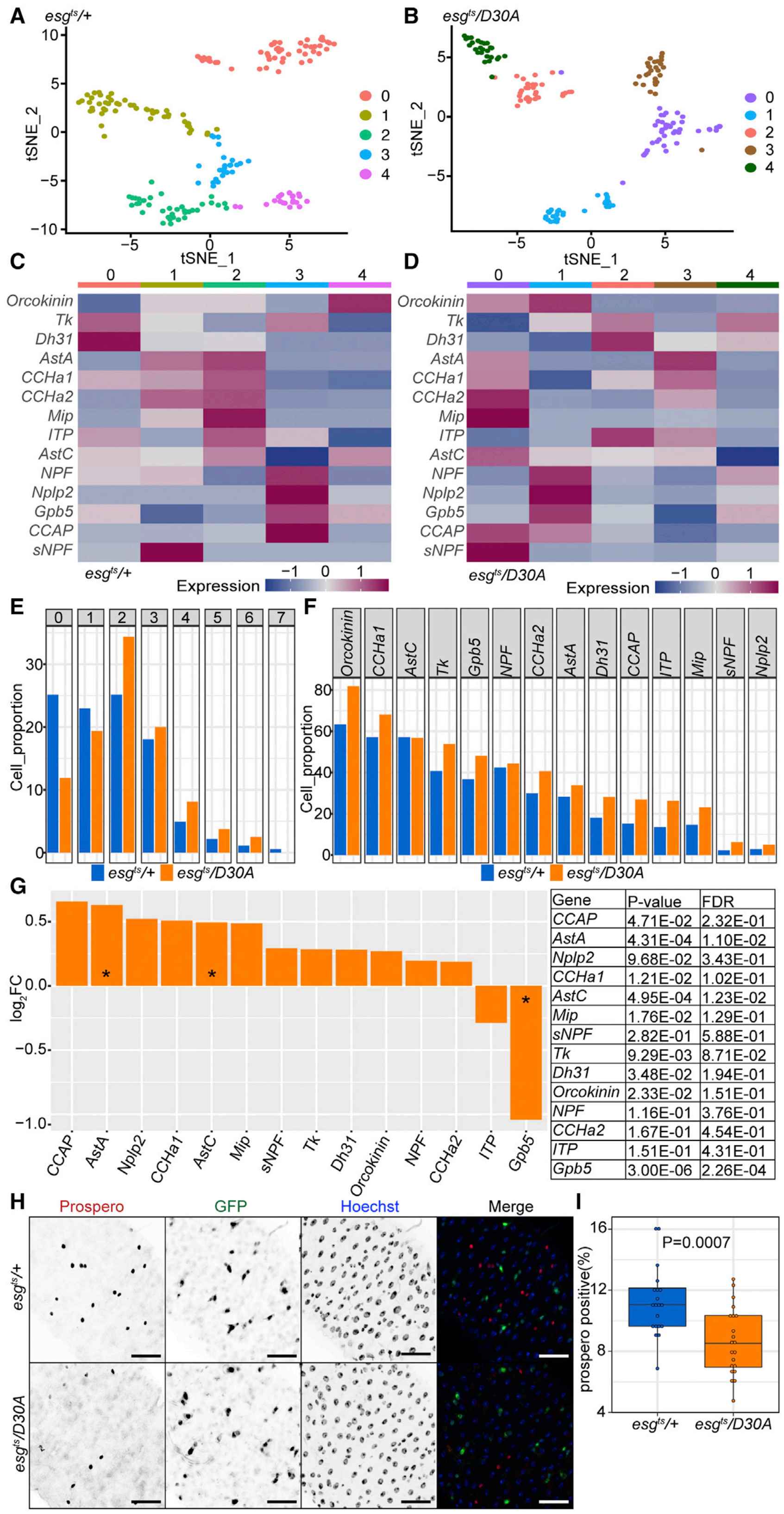

Gene	P-value	FDR
CCAP	4.71E-02	2.32E-01
AstA	4.31E-04	1.10E-02
Nplp2	9.68E-02	3.43E-01
CCHa1	1.21E-02	1.02E-01
AstC	4.95E-04	1.23E-02
Mip	1.76E-02	1.29E-01
sNPF	2.82E-01	5.88E-01
Tk	9.29E-03	8.71E-02
Dh31	3.48E-02	1.94E-01
Orcokinin	2.33E-02	1.51E-01
NPF	1.16E-01	3.76E-01
CCHa2	1.67E-01	4.54E-01
ITP	1.51E-01	4.31E-01
Gpb5	3.00E-06	2.26E-04

Figure 5. Inhibition of progenitor-cell IMD affects generation of mature enteroendocrine cells

(A and B) t-distributed stochastic neighbor embedding (tSNE) plot visualizing subsets of *prospero*-positive enteroendocrine cells in *esg*^{ts}/+ (A) and *esg*^{ts}*/D30A* intestines (B) based on the expression of marker genes. Cells are color-coded by cell subset.

(C and D) Heatmap showing relative expression of fourteen peptide hormones in each enteroendocrine cell subset in *esg*^{ts}/+ (C) and *esg*^{ts}*/D30A* (D) intestines.

(E) Quantification of the percentage of enteroendocrine cells that express the indicated numbers of peptide hormones. Genotypes are color-coded as indicated.

(F) Quantification of the percentage of enteroendocrine cells that express the indicated peptide hormone. Genotypes are color-coded as indicated.

(G) Quantification of the relative expression of each peptide hormone in isolated posterior midguts from *esg*^{ts}*/D30A* flies relative to *esg*^{ts}/+ flies based on bulk RNA-seq analysis.

(H) Visualization of Prospero, GFP, and DNA (Hoechst) in intestines of 10-day-old *esg*^{ts}/+ and *esg*^{ts}*/D30A* flies. Scale bars represent 25 μm.

(I) Quantification of the percentage of intestinal epithelial cells that express the enteroendocrine cell marker *prospero* in the intestines of 10-day-old flies as indicated. Statistical significance was calculated using Student's t test. For *esg*^{ts}/+ flies n = 20, and for *esg*^{ts}*/D30A* flies n = 21.

2018). There are conflicting data on consequences of IMD inactivation in progenitors, with one study suggesting decreased proliferation (Wang et al., 2019), and a separate study indicating increased proliferation (Wang et al., 2013). We found that progenitor-specific inactivation of IMD diminished ISC proliferation, impaired age-dependent accumulation of *esg*-positive and Dl-positive progenitors, elevated the number of *Su*(*H*)-positive enteroblasts, and resulted in differentiation defects that included both fewer EE cells and shifts in gene-expression patterns associated with EC subtypes. Thus, our work suggests that progenitor-specific immunity contributes to epithelial homeostasis in flies and raises several questions about effects of progenitor-cell IMD on the adult gut.

Which progenitor cell requires IMD to regulate differentiation? The fly progenitor compartment consists of undifferentiated ISCs and post-mitotic enteroblasts that are committed to EC cell fate. Progenitors express IMD-pathway components, and genomic studies, including data presented here, show that ISCs and enteroblasts have highly similar gene-expression profiles (Dutta et al., 2015; Hung et al., 2020), suggesting that both cell types are likely equally competent at IMD activation. ISCs are basally situated within the midgut epithelium and are not expected to make frequent, direct contacts with the intestinal lumen. Enteroblasts are the apical daughters of ISC divisions that occur at oblique angles to the basement membrane. Thus, it seems more plausible that enteroblasts directly contact the lumen where they can detect PGN. However, it is important to note that gut-derived PGN is not strictly confined to the intestinal lumen in flies or vertebrates. PGN crosses the epithelial barrier, even in the absence of detectable breaches, and several mechanisms are in place to prevent accumulation of PGN in the fly hemolymph (Capo et al., 2017; Gendrin et al., 2009; Paredes et al., 2011; Troha et al., 2019; Zaidman-Rémy et al., 2006). Thus, we cannot exclude the possibility that passive, or active, transport mechanisms allow diffusion of PGN across the gut barrier to ISCs, which in turn activate IMD and modulate differentiation responses in the hosts. In this regard, we consider it interesting that several vertebrate pattern-recognition receptors have cell-type-specific, apicobasal distribution patterns. For instance, TLR4 is enriched apically in villi and basolaterally in crypts of the human colon (Fusunyan et al., 2001). Furthermore, apical stimulation of TLR9 promotes JNK activation, whereas basolateral stimulation of TLR9 leads to NF-kB activation, and IL-8 production (Lee et al., 2006). In future assays, it will be interesting to determine if basolateral detection of PGN influences fate choices within the fly progenitor compartment.

Is progenitor-specific IMD necessary to generate growth-regulatory ECs? Inhibition of progenitor-cell IMD did not block IMD-dependent immune responses in ECs, confirming that the phenotypes reported here are not a consequence of ImdD30A perdurance in differentiated epithelia. However, we also found that inhibition of IMD in progenitors had consequences for epithelial differentiation, including effects on gene-expression patterns within mature ECs. As ECs produce paracrine regulators of progenitor proliferation and differentiation, we cannot exclude the possibility that blocking IMD in progenitors disrupts enteroblast differentiation in a manner that modifies the ability of ECs to transduce growth and differentiation cues to progenitors. This may be particularly important in the context of epithelial damage, where secreted factors from dying ECs accelerate ISC proliferation to maintain the epithelial barrier and regenerate a mature gut. In this scenario, IMD activity in progenitors is important to establish homeostatic intercellular communications between ECs and the progenitor compartment, and loss of progenitor-cell IMD interrupts a developmental loop between progenitors and ECs. Consistent with requirements for IMD in the control of epithelial differentiation, we noticed that IMD in progenitors affected the generation of mature EE cells. Specifically, inhibition of progenitor-cell IMD led to a decline in EE numbers but a general increase in the expression of peptide hormones, possibly as a compensatory mechanism. Notably, flies raised in an axenic environment have fewer enteroblasts and more EE cells (Broderick et al., 2014), indicating non-overlapping contributions of progenitor-cell immunity and gut microbes to developmental trajectories of ISCs.

Does IMD have regional effects on intestinal progenitors? Intestines are functionally specialized along the rostro-caudal axis, with distinct partitions governing various aspects of food digestion and absorption. IMD also displays clear signs of regional specialization. The foregut is characterized by enriched expression of PGRP-LC, and Rel regulates expression of chitin-binding proteins that contribute to peritrophic matrix construction (Buchon et al., 2009b; Neyen et al., 2012). In the midgut, PGN detection primarily relies on PGRP-LE, particularly in the posterior midgut (Bosco-Drayon et al., 2012; Neyen et al., 2012). Activation of IMD in the anterior midgut results in expression of antimicrobial peptides that protect the fly from ingested microbes (Buchon et al., 2013b). In contrast, IMD activation leads to delamination of damaged cells in the midgut of flies infected with pathogenic bacteria, most notably in the R4 region of the posterior midgut (Zhai et al., 2018). In the posterior midgut, the transcription factor caudal prevents IMD-dependent antimicrobial peptide expression (Ryu et al., 2008). Instead, IMD induces expression of molecules that dampen immune signaling, including the PGRP-LC inhibitor *pirk*, and amidases that scavenge PGN (Bosco-Drayon et al., 2012). As a result, posterior midgut IMD establishes a tolerogenic environment for commensal bacteria. Notably, loss of IMD pathway inhibitors or expression

of PGRP-LC in ECs, increases proliferation in the posterior midgut (Paredes et al., 2011; Zhai et al., 2018), raising the possibility that suppression of posterior midgut IMD activity is required to prevent excess proliferation in the absence of infection. With a large collection of genetic reagents, and accessible genomic methods, the fly is an excellent system to systematically characterize regional effects of immune responses on progenitor-cell function. Given the evolutionary conservation of immune responses, we believe the findings reported in this study to be of relevance for understanding fundamental principles of immune-regulated intestinal homeostasis.

EXPERIMENTAL PROCEDURES

Fly husbandry

Flies were raised on corn meal medium (Nutri-Fly Bloomington Formulation, https://bdsc.indiana.edu/information/recipes/bloomfood.html; Genesse Scientific) at 18°C or 25°C. All experimental flies were adult virgin females kept under a 12h:12h light:dark cycle and maintained at 18°C during collection then shifted to 29°C to express downstream genes as indicated. We used w^{1118} as a wild-type strain, backcrossed *UAS-imdD30A* transgenic lines into the w^{1118} background for eight generations prior to use, and used standard husbandry methods to ensure that esg^{ts} (*esg-GAL4, tub-GAL80*^{ts}, *UAS-GFP*) flies had the same first and third chromosomes as our w^{1118} line. Fly lines used in this study were: *w;esg-GAL4,tub-GAL80*^{ts}*,UAS-GFP* (referred to as esg^{ts}); *UAS-FADD*^{RNAi} (VDRC ID# 7926); w^{1118} (VDRC ID# 60000); *w;esg-GAL4,UAS-CFP, Su(H)-GFP;tubGal80*^{ts} (esg^{ts}*,UAS-CFP,Su(H)-GFP*); *GS 5961* (Mathur et al., 2010); *dpt-GFP, esg-GAL4, tubGAL80ts, UAS-GFP;UAS-flp, Act> CD2>GAL4* (referred to as $esg^{F/O}$); *Esg[ts], Su(H) Gal80* (referred to as ISC^{ts}); *Su(H)GBE-Gal4*^{ts} (referred to as $Su(H)^{ts}$); *PGRP-LE RNAi* (VDRC ID# 108199); *PGRP-LC RNAi* (VDRC ID# 101636); *Rel-RNAi* (VDRC ID# 49413); and *40D-UAS* (control for VDRC KK lines, VDRC ID# 60101) . To induce GFP-marked mitotic clones using the $esg^{F/O}$ system, flies of the indicated genotype were raised at 18°C for 3 days after eclosion, shifted to 29°C for 16 h, then raised at 25°C for an additional 9 days.

Data availability

The accession number for the gene expression data reported in this paper is GEO: SuperSeries GSE141897 (GSE171001 and GSE141896). The accession number for the Single cell gene expression data reported in this paper is Broad Institute Single Cell Portal: for $esg^{ts/+}$ flies (https://singlecell.broadinstitute.org/single_cell/study/SCP1696/single-cell-expression-data-for-d-melanogasterwild-type-intestines) and for $esg^{ts/D30A}$ flies (https://singlecell.broadinstitute.org/single_cell/study/SCP1699/single-cell-expression-data-for-d-melanogasterintestines-with-immune-deficient-progenitor-cells#study-summary).

SUPPLEMENTAL INFORMATION

Supplemental information can be found online at https://doi.org/10.1016/j.stemcr.2022.02.009.

AUTHOR CONTRIBUTIONS

Conceptualization: M.S. and E.F.; methodology: M.S. and E.F.; formal analysis: M.S., M.F., and E.F.; investigation: M.S., M.F., R.J.W., L.O.J., and K.P.; data curation: M.S. and M.F.; writing – original draft: M.S. and E.F.; writing – review & editing: M.S., M.F., and E.F.; visualization: M.S. and M.F.; supervision: E.F.; project administration: E.F.; funding acquisition: E.F.

CONFLICTS OF INTEREST

The authors declare no competing interests.

ACKNOWLEDGMENTS

Drosophila lines were provided by Drs. Shelagh Campbell, Lucy O'Brien, Bruce Edgar, Heinrich Jasper, and Bruno Lemaitre. We acknowledge microscopy support from Dr. Steven Ogg and Gregory Plummer at the Faculty of Medicine and Dentistry Imaging core, flow cytometry support from Dr. Aja Rieger at the Faculty of Medicine and Dentistry Flow Cytometry Core, and support with single-cell library preparation from Dr. Joaquin Lopez-Orozco. We are grateful to Dr. David Fast for assistance with analysis of the RNA-seq data. The authors wish to thank Kin Chan at the Network Biology Collaborative Centre for the RNA-seq service. Network Biology Collaborative Centre is a facility supported by Canada Foundation for Innovation, the Ontarian government, and Genome Canada and Ontario Genomics (OGI-139). This work was supported by a grant from the Canadian Institute of Health Research (grant no. PJT 159604). M.S. was supported by Basic Science Research Program through the National Research Foundation of Korea (NRF) funded by the Ministry of Education (2020R1A6A3A0303955511). M.F. has funding through Alberta Innovates Graduate Student Scholarships and an NSERC PGS-D.

Received: April 29, 2021
Revised: February 16, 2022
Accepted: February 16, 2022
Published: March 17, 2022

REFERENCES

Bardin, A.J., Perdigoto, C.N., Southall, T.D., Brand, A.H., and Schweisguth, F. (2010). Transcriptional control of stem cell maintenance in the Drosophila intestine. Development *137*, 705–714. https://doi.org/10.1242/dev.039404.

Barker, N. (2014). Adult intestinal stem cells: critical drivers of epithelial homeostasis and regeneration. Nat. Rev. Mol. Cell Biol. *15*, 19–33. https://doi.org/10.1038/nrm3721.

Beehler-Evans, R., and Micchelli, C.A. (2015). Generation of enteroendocrine cell diversity in midgut stem cell lineages. Dev. Camb. Engl. *142*, 654–664. https://doi.org/10.1242/dev.114959.

Biteau, B., and Jasper, H. (2014). Slit/Robo signaling regulates cell fate decisions in the intestinal stem cell lineage of Drosophila. Cell Rep *7*, 1867–1875. https://doi.org/10.1016/j.celrep.2014.05.024.

Biton, M., Haber, A.L., Rogel, N., Burgin, G., Beyaz, S., Schnell, A., Ashenberg, O., Su, C.-W., Smillie, C., Shekhar, K., et al. (2018). T

Helper Cell Cytokines Modulate Intestinal Stem Cell Renewal and Differentiation. Cell *175*, 1307–1320.e22. https://doi.org/10.1016/j.cell.2018.10.008.

Bosco-Drayon, V., Poidevin, M., Boneca, I.G., Narbonne-Reveau, K., Royet, J., and Charroux, B. (2012). Peptidoglycan Sensing by the Receptor PGRP-LE in the Drosophila Gut Induces Immune Responses to Infectious Bacteria and Tolerance to Microbiota. Cell Host Microbe *12*, 153–165. https://doi.org/10.1016/j.chom.2012.06.002.

Broderick, N.A., Buchon, N., and Lemaitre, B. (2014). Microbiota-induced changes in drosophila melanogaster host gene expression and gut morphology. mBio *5*. e01117–01114. https://doi.org/10.1128/mBio.01117-14.

Brugman, S. (2016). The zebrafish as a model to study intestinal inflammation. Dev. Comp. Immunol. *64*, 82–92. https://doi.org/10.1016/j.dci.2016.02.020.

Buchon, N., Broderick, N.A., Chakrabarti, S., and Lemaitre, B. (2009a). Invasive and indigenous microbiota impact intestinal stem cell activity through multiple pathways in Drosophila. Genes Dev *23*, 2333–2344. https://doi.org/10.1101/gad.1827009.

Buchon, N., Broderick, N.A., and Lemaitre, B. (2013a). Gut homeostasis in a microbial world: insights from Drosophila melanogaster. Nat. Rev. Microbiol. *11*, 615–626. https://doi.org/10.1038/nrmicro3074.

Buchon, N., Broderick, N.A., Poidevin, M., Pradervand, S., and Lemaitre, B. (2009b). Drosophila intestinal response to bacterial infection: activation of host defense and stem cell proliferation. Cell Host Microbe *5*, 200–211. https://doi.org/10.1016/j.chom.2009.01.003.

Buchon, N., Osman, D., David, F.P.A., Fang, H.Y., Boquete, J.-P., Deplancke, B., and Lemaitre, B. (2013b). Morphological and molecular characterization of adult midgut compartmentalization in Drosophila. Cell Rep *3*, 1725–1738. https://doi.org/10.1016/j.celrep.2013.04.001.

Capo, F., Chaduli, D., Viallat-Lieutaud, A., Charroux, B., and Royet, J. (2017). Oligopeptide Transporters of the SLC15 Family Are Dispensable for Peptidoglycan Sensing and Transport in Drosophila. J. Innate Immun. *9*, 483–492. https://doi.org/10.1159/000475771.

Casali, A., and Batlle, E. (2009). Intestinal stem cells in mammals and Drosophila. Cell Stem Cell *4*, 124–127. https://doi.org/10.1016/j.stem.2009.01.009.

Couturier-Maillard, A., Secher, T., Rehman, A., Normand, S., Arcangelis, A.D., Haesler, R., Huot, L., Grandjean, T., Bressenot, A., Delanoye-Crespin, A., et al. (2013). NOD2-mediated dysbiosis predisposes mice to transmissible colitis and colorectal cancer. J. Clin. Invest. *123*, 700–711. https://doi.org/10.1172/JCI62236.

Dutta, D., Dobson, A.J., Houtz, P.L., Gläßer, C., Revah, J., Korzelius, J., Patel, P.H., Edgar, B.A., and Buchon, N. (2015). Regional Cell-Specific Transcriptome Mapping Reveals Regulatory Complexity in the Adult Drosophila Midgut. Cell Rep *12*, 346–358. https://doi.org/10.1016/j.celrep.2015.06.009.

Fast, D., Petkau, K., Ferguson, M., Shin, M., Galenza, A., Kostiuk, B., Pukatzki, S., and Foley, E. (2020). Vibrio cholerae-Symbiont Interactions Inhibit Intestinal Repair in Drosophila. Cell Rep *30*, 1088–1100.e5. https://doi.org/10.1016/j.celrep.2019.12.094.

Ferguson, M., and Foley, E. (2021). Microbial recognition regulates intestinal epithelial growth in homeostasis and disease. FEBS J https://doi.org/10.1111/febs.15910.

Fu, Y.-Y., Egorova, A., Sobieski, C., Kuttiyara, J., Calafiore, M., Takashima, S., Clevers, H., and Hanash, A.M. (2019). T Cell Recruitment to the Intestinal Stem Cell Compartment Drives Immune-Mediated Intestinal Damage after Allogeneic Transplantation. Immunity *51*, 90–103.e3. https://doi.org/10.1016/j.immuni.2019.06.003.

Fukata, M., Chen, A., Klepper, A., Krishnareddy, S., Vamadevan, A.S., Thomas, L.S., Xu, R., Inoue, H., Arditi, M., Dannenberg, A.J., et al. (2006). Cox-2 is regulated by Toll-like receptor-4 (TLR4) signaling: Role in proliferation and apoptosis in the intestine. Gastroenterology *131*, 862–877. https://doi.org/10.1053/j.gastro.2006.06.017.

Fusunyan, R.D., Nanthakumar, N.N., Baldeon, M.E., and Walker, W.A. (2001). Evidence for an Innate Immune Response in the Immature Human Intestine: Toll-Like Receptors on Fetal Enterocytes. Pediatr. Res. *49*, 589–593. https://doi.org/10.1203/00006450-200104000-00023.

Gendrin, M., Welchman, D.P., Poidevin, M., Hervé, M., and Lemaitre, B. (2009). Long-Range Activation of Systemic Immunity through Peptidoglycan Diffusion in Drosophila. PLOS Pathog *5*, e1000694. https://doi.org/10.1371/journal.ppat.1000694.

Guo, L., Karpac, J., Tran, S.L., and Jasper, H. (2014). PGRP-SC2 promotes gut immune homeostasis to limit commensal dysbiosis and extend lifespan. Cell *156*, 109–122. https://doi.org/10.1016/j.cell.2013.12.018.

Guo, X., Yin, C., Yang, F., Zhang, Y., Huang, H., Wang, J., Deng, B., Cai, T., Rao, Y., and Xi, R. (2019). The Cellular Diversity and Transcription Factor Code of Drosophila Enteroendocrine Cells. Cell Rep *29*, 4172–4185.e5. https://doi.org/10.1016/j.celrep.2019.11.048.

Guo, Z., and Ohlstein, B. (2015). Bidirectional Notch signaling regulates Drosophila intestinal stem cell multipotency. Science *350*. https://doi.org/10.1126/science.aab0988.

Hsu, D., Fukata, M., Hernandez, Y.G., Sotolongo, J.P., Goo, T., Maki, J., Hayes, L.A., Ungaro, R.C., Chen, A., Breglio, K.J., et al. (2010). Toll-like receptor 4 differentially regulates epidermal growth factor-related growth factors in response to intestinal mucosal injury. Lab. Investig. J. Tech. Methods Pathol. *90*, 1295–1305. https://doi.org/10.1038/labinvest.2010.100.

Hugot, J.P., Chamaillard, M., Zouali, H., Lesage, S., Cézard, J.P., Belaiche, J., Almer, S., Tysk, C., O'Morain, C.A., Gassull, M., et al. (2001). Association of NOD2 leucine-rich repeat variants with susceptibility to Crohn's disease. Nature *411*, 599–603. https://doi.org/10.1038/35079107.

Hung, R.-J., Hu, Y., Kirchner, R., Liu, Y., Xu, C., Comjean, A., Tattikota, S.G., Li, F., Song, W., Sui, S.H., et al. (2020). A cell atlas of the adult Drosophila midgut. Proc. Natl. Acad. Sci. *117*, 1514–1523. https://doi.org/10.1073/pnas.1916820117.

Kamareddine, L., Robins, W.P., Berkey, C.D., Mekalanos, J.J., and Watnick, P.I. (2018). The Drosophila Immune Deficiency Pathway

Modulates Enteroendocrine Function and Host Metabolism. Cell Metab *28*, 449–462.e5. https://doi.org/10.1016/j.cmet.2018.05.026.

Kim, C.-H., Paik, D., Rus, F., and Silverman, N. (2014). The caspase-8 homolog Dredd cleaves Imd and Relish but is not inhibited by p35. J. Biol. Chem. *289*, 20092–20101. https://doi.org/10.1074/jbc.M113.544841.

Lee, J., Mo, J.-H., Katakura, K., Alkalay, I., Rucker, A.N., Liu, Y.-T., Lee, H.-K., Shen, C., Cojocaru, G., Shenouda, S., et al. (2006). Maintenance of colonic homeostasis by distinctive apical TLR9 signalling in intestinal epithelial cells. Nat. Cell Biol. *8*, 1327–1336. https://doi.org/10.1038/ncb1500.

Leulier, F., Rodriguez, A., Khush, R.S., Abrams, J.M., and Lemaitre, B. (2000). The Drosophila caspase Dredd is required to resist gram-negative bacterial infection. EMBO Rep *1*, 353–358. https://doi.org/10.1093/embo-reports/kvd073.

Levy, A., Stedman, A., Deutsch, E., Donnadieu, F., Virgin, H.W., Sansonetti, P.J., and Nigro, G. (2020). Innate immune receptor NOD2 mediates LGR5+ intestinal stem cell protection against ROS cytotoxicity via mitophagy stimulation. Proc. Natl. Acad. Sci. *117*, 1994–2003. https://doi.org/10.1073/pnas.1902788117.

Lickwar, C.R., Camp, J.G., Weiser, M., Cocchiaro, J.L., Kingsley, D.M., Furey, T.S., Sheikh, S.Z., and Rawls, J.F. (2017). Genomic dissection of conserved transcriptional regulation in intestinal epithelial cells. PLoS Biol *15*, e2002054. https://doi.org/10.1371/journal.pbio.2002054.

Mathur, D, Bost, A, Driver, I, and Ohlstein, B (2010). A transient niche regulates the specification of *Drosophila* intestinal stem cells. Science *327*, 210–213. https://doi.org/10.1126/science.1181958.

Micchelli, C.A., and Perrimon, N. (2006). Evidence that stem cells reside in the adult Drosophila midgut epithelium. Nature *439*, 475–479. https://doi.org/10.1038/nature04371.

Miguel-Aliaga, I., Jasper, H., and Lemaitre, B. (2018). Anatomy and Physiology of the Digestive Tract of Drosophila melanogaster. Genetics *210*, 357–396. https://doi.org/10.1534/genetics.118.300224.

Myllymäki, H., Valanne, S., and Rämet, M. (2014). The Drosophila Imd Signaling Pathway. J. Immunol. *192*, 3455–3462. https://doi.org/10.4049/jimmunol.1303309.

Naito, T., Mulet, C., De Castro, C., Molinaro, A., Saffarian, A., Nigro, G., Bérard, M., Clerc, M., Pedersen, A.B., Sansonetti, P.J., et al. (2017). Lipopolysaccharide from Crypt-Specific Core Microbiota Modulates the Colonic Epithelial Proliferation-to-Differentiation Balance. mBio *8*. e01680–17. https://doi.org/10.1128/mBio.01680-17.

Neal, M.D., Sodhi, C.P., Jia, H., Dyer, M., Egan, C.E., Yazji, I., Good, M., Afrazi, A., Marino, R., Slagle, D., et al. (2012). Toll-like Receptor 4 Is Expressed on Intestinal Stem Cells and Regulates Their Proliferation and Apoptosis via the p53 Up-regulated Modulator of Apoptosis. J. Biol. Chem. *287*, 37296–37308. https://doi.org/10.1074/jbc.M112.375881.

Neyen, C., Poidevin, M., Roussel, A., and Lemaitre, B. (2012). Tissue- and ligand-specific sensing of gram-negative infection in drosophila by PGRP-LC isoforms and PGRP-LE. J. Immunol. *189*, 1886–1897. https://doi.org/10.4049/jimmunol.1201022.

Nguyen, T.L.A., Vieira-Silva, S., Liston, A., and Raes, J. (2015). How informative is the mouse for human gut microbiota research? Dis. Model. Mech. *8*, 1–16. https://doi.org/10.1242/dmm.017400.

Nigro, G., Rossi, R., Commere, P.-H., Jay, P., and Sansonetti, P.J. (2014). The cytosolic bacterial peptidoglycan sensor Nod2 affords stem cell protection and links microbes to gut epithelial regeneration. Cell Host Microbe *15*, 792–798. https://doi.org/10.1016/j.chom.2014.05.003.

Ogura, Y., Bonen, D.K., Inohara, N., Nicolae, D.L., Chen, F.F., Ramos, R., Britton, H., Moran, T., Karaliuskas, R., Duerr, R.H., et al. (2001). A frameshift mutation in NOD2 associated with susceptibility to Crohn's disease. Nature *411*, 603–606. https://doi.org/10.1038/35079114.

Ohlstein, B., and Spradling, A. (2007). Multipotent Drosophila intestinal stem cells specify daughter cell fates by differential notch signaling. Science *315*, 988–992. https://doi.org/10.1126/science.1136606.

Ohlstein, B., and Spradling, A. (2006). The adult Drosophila posterior midgut is maintained by pluripotent stem cells. Nature *439*, 470–474. https://doi.org/10.1038/nature04333.

Paquette, N., Broemer, M., Aggarwal, K., Chen, L., Husson, M., Ertürk-Hasdemir, D., Reichhart, J.-M., Meier, P., and Silverman, N. (2010). Caspase-mediated cleavage, IAP binding, and ubiquitination: linking three mechanisms crucial for Drosophila NF-kappaB signaling. Mol. Cell *37*, 172–182. https://doi.org/10.1016/j.molcel.2009.12.036.

Paredes, J.C., Welchman, D.P., Poidevin, M., and Lemaitre, B. (2011). Negative Regulation by Amidase PGRPs Shapes the Drosophila Antibacterial Response and Protects the Fly from Innocuous Infection. Immunity *35*, 770–779. https://doi.org/10.1016/j.immuni.2011.09.018.

Petkau, K., Ferguson, M., Guntermann, S., and Foley, E. (2017). Constitutive Immune Activity Promotes Tumorigenesis in Drosophila Intestinal Progenitor Cells. Cell Rep *20*, 1784–1793. https://doi.org/10.1016/j.celrep.2017.07.078.

Price, A.E., Shamardani, K., Lugo, K.A., Deguine, J., Roberts, A.W., Lee, B.L., and Barton, G.M. (2018). A Map of Toll-like Receptor Expression in the Intestinal Epithelium Reveals Distinct Spatial, Cell Type-Specific, and Temporal Patterns. Immunity *49*, 560–575.e6. https://doi.org/10.1016/j.immuni.2018.07.016.

Ren, C., Webster, P., Finkel, S.E., and Tower, J. (2007). Increased internal and external bacterial load during Drosophila aging without life-span trade-off. Cell Metab *6*, 144–152. https://doi.org/10.1016/j.cmet.2007.06.006.

Ryu, J.-H., Kim, S.-H., Lee, H.-Y., Bai, J.Y., Nam, Y.-D., Bae, J.-W., Lee, D.G., Shin, S.C., Ha, E.-M., and Lee, W.-J. (2008). Innate immune homeostasis by the homeobox gene caudal and commensal-gut mutualism in Drosophila. Science *319*, 777–782. https://doi.org/10.1126/science.1149357.

Sancho, R., Cremona, C.A., and Behrens, A. (2015). Stem cell and progenitor fate in the mammalian intestine: Notch and lateral inhibition in homeostasis and disease. EMBO Rep *16*, 571–581. https://doi.org/10.15252/embr.201540188.

Spit, M., Koo, B.-K., and Maurice, M.M. (2018). Tales from the crypt: intestinal niche signals in tissue renewal, plasticity and cancer. Open Biol *8*, 180120. https://doi.org/10.1098/rsob.180120.

Stöven, S., Ando, I., Kadalayil, L., Engström, Y., and Hultmark, D. (2000). Activation of the Drosophila NF-kappaB factor Relish by rapid endoproteolytic cleavage. EMBO Rep *1*, 347–352. https://doi.org/10.1093/embo-reports/kvd072.

Stoven, S., Silverman, N., Junell, A., Hedengren-Olcott, M., Erturk, D., Engstrom, Y., Maniatis, T., and Hultmark, D. (2003). Caspase-mediated processing of the Drosophila NF-kappaB factor Relish. Proc. Natl. Acad. Sci. U S A *100*, 5991–5996. https://doi.org/10.1073/pnas.1035902100.

Takashima, S., Martin, M.L., Jansen, S.A., Fu, Y., Bos, J., Chandra, D., O'Connor, M.H., Mertelsmann, A.M., Vinci, P., Kuttiyara, J., et al. (2019). T cell-derived interferon-γ programs stem cell death in immune-mediated intestinal damage. Sci. Immunol. *4*. https://doi.org/10.1126/sciimmunol.aay8556.

Troha, K., Nagy, P., Pivovar, A., Lazzaro, B.P., Hartley, P.S., and Buchon, N. (2019). Nephrocytes Remove Microbiota-Derived Peptidoglycan from Systemic Circulation to Maintain Immune Homeostasis. Immunity *51*, 625–637.e3. https://doi.org/10.1016/j.immuni.2019.08.020.

Vooijs, M., Liu, Z., and Kopan, R. (2011). Notch: architect, landscaper, and guardian of the intestine. Gastroenterology *141*, 448–459. https://doi.org/10.1053/j.gastro.2011.06.003.

Wallace, K.N., Akhter, S., Smith, E.M., Lorent, K., and Pack, M. (2005). Intestinal growth and differentiation in zebrafish. Mech. Dev. *122*, 157–173. https://doi.org/10.1016/j.mod.2004.10.009.

Wang, L., Sloan, M.A., and Ligoxygakis, P. (2019). Intestinal NF-κB and STAT signalling is important for uptake and clearance in a Drosophila-Herpetomonas interaction model. PLOS Genet *15*, e1007931. https://doi.org/10.1371/journal.pgen.1007931.

Wang, Z., Hang, S., Purdy, A.E., and Watnick, P.I. (2013). Mutations in the IMD Pathway and Mustard Counter Vibrio cholerae Suppression of Intestinal Stem Cell Division in Drosophila. mBio *4*. https://doi.org/10.1128/mBio.00337-13.

Xu, N., Wang, S.Q., Tan, D., Gao, Y., Lin, G., and Xi, R. (2011). EGFR, Wingless and JAK/STAT signaling cooperatively maintain Drosophila intestinal stem cells. Dev. Biol. *354*, 31–43. https://doi.org/10.1016/j.ydbio.2011.03.018.

Zaidman-Rémy, A., Hervé, M., Poidevin, M., Pili-Floury, S., Kim, M.-S., Blanot, D., Oh, B.-H., Ueda, R., Mengin-Lecreulx, D., and Lemaitre, B. (2006). The Drosophila amidase PGRP-LB modulates the immune response to bacterial infection. Immunity *24*, 463–473. https://doi.org/10.1016/j.immuni.2006.02.012.

Zeng, X., and Hou, S.X. (2015). Enteroendocrine cells are generated from stem cells through a distinct progenitor in the adult Drosophila posterior midgut. Development *142*, 644–653. https://doi.org/10.1242/dev.113357.

Zhai, Z., Boquete, J.-P., and Lemaitre, B. (2018). Cell-Specific Imd-NF-κB Responses Enable Simultaneous Antibacterial Immunity and Intestinal Epithelial Cell Shedding upon Bacterial Infection. Immunity *48*, 897–910.e7. https://doi.org/10.1016/j.immuni.2018.04.010.

Stem Cell Reports, Volume *17*

Supplemental Information

Immune regulation of intestinal-stem-cell function in *Drosophila*

Minjeong Shin, Meghan Ferguson, Reegan J. Willms, Lena O. Jones, Kristina Petkau, and Edan Foley

SUPPLEMENTAL FIGURES

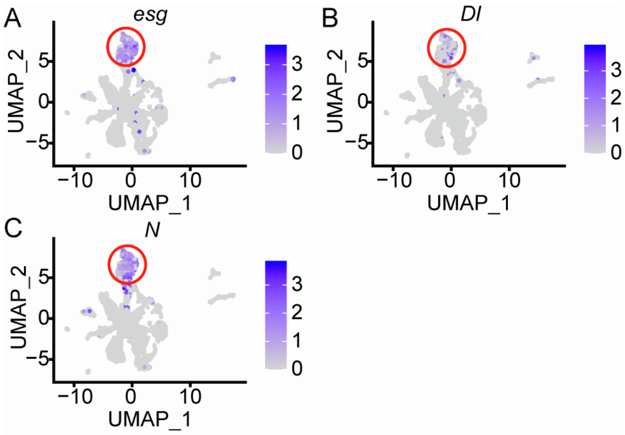

Figure S1

Figure S1. **Expression pattern of *Drosophila* progenitor cell markers, Related to Figure 1.**
Feature plots showing expression of *Drosophila* progenitor cell markers *escargot* (*esg*), *Delta (Dl*), and *Notch* (*N*) in an unsupervised UMAP prepared with single-cell expression data from adult *esg*^{ts}/+ female *Drosophila* midguts. Progenitor cells are indicated with a red circle.

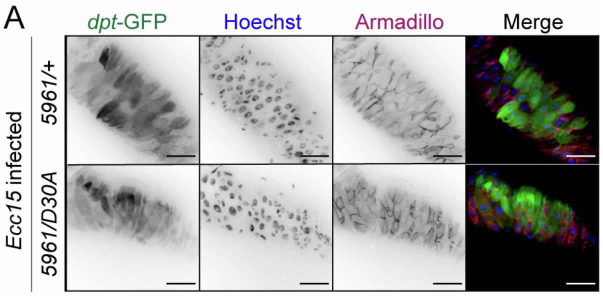

B

Genotype	Control	D30A
Sample size	9	10
% GFP +ive	100	100

Control: *GS 5961/+;dpt-GFP*

D30A: *GS 5961/D30A;dpt-GFP*

Supplementary Figure 2

Figure S2. Inhibition of IMD in progenitor cell does not affect IMD activity in differentiated progeny, related to Figure 1.

A: Visualization of the IMD pathway reporter *diptericin:GFP* (*dpt*-GFP), DNA (Hoechst), and the beta-catenin ortholog Armadillo in intestines of adult female *Drosophila* infected overnight with pathogenic *Ecc15*. The esg^{ts} line used in the rest of the study marks progenitor cells with GFP, preventing us from unambiguously identifying cells that expressed GFP under control of the *dpt* promoter in an esg^{ts} background. Therefore, we used the *GS5961* gene switch fly line for RU486-dependent induction of the GAL4 transcription factor in intestinal progenitor cells in this experiment. In the upper row, we visualized *dpt*-GFP expression in *GS5961/+* (*5961/+*) flies, and in the lower row, we visualized *dpt*-GFP expression in *GS5961/UAS-imdD30A* (*5961/D30A*) flies. Both lines were treated with RU486 for 48h prior to infection. Scale bars represent 25μm. **B:** Quantification of fly guts of the indicated genotypes that expressed *dpt*-GFP in enterocytes after infection with *Ecc15.* Expression of imdD30A in progenitors (*D30A*) did not prevent infection-mediated activation of IMD responses in mature enterocytes.

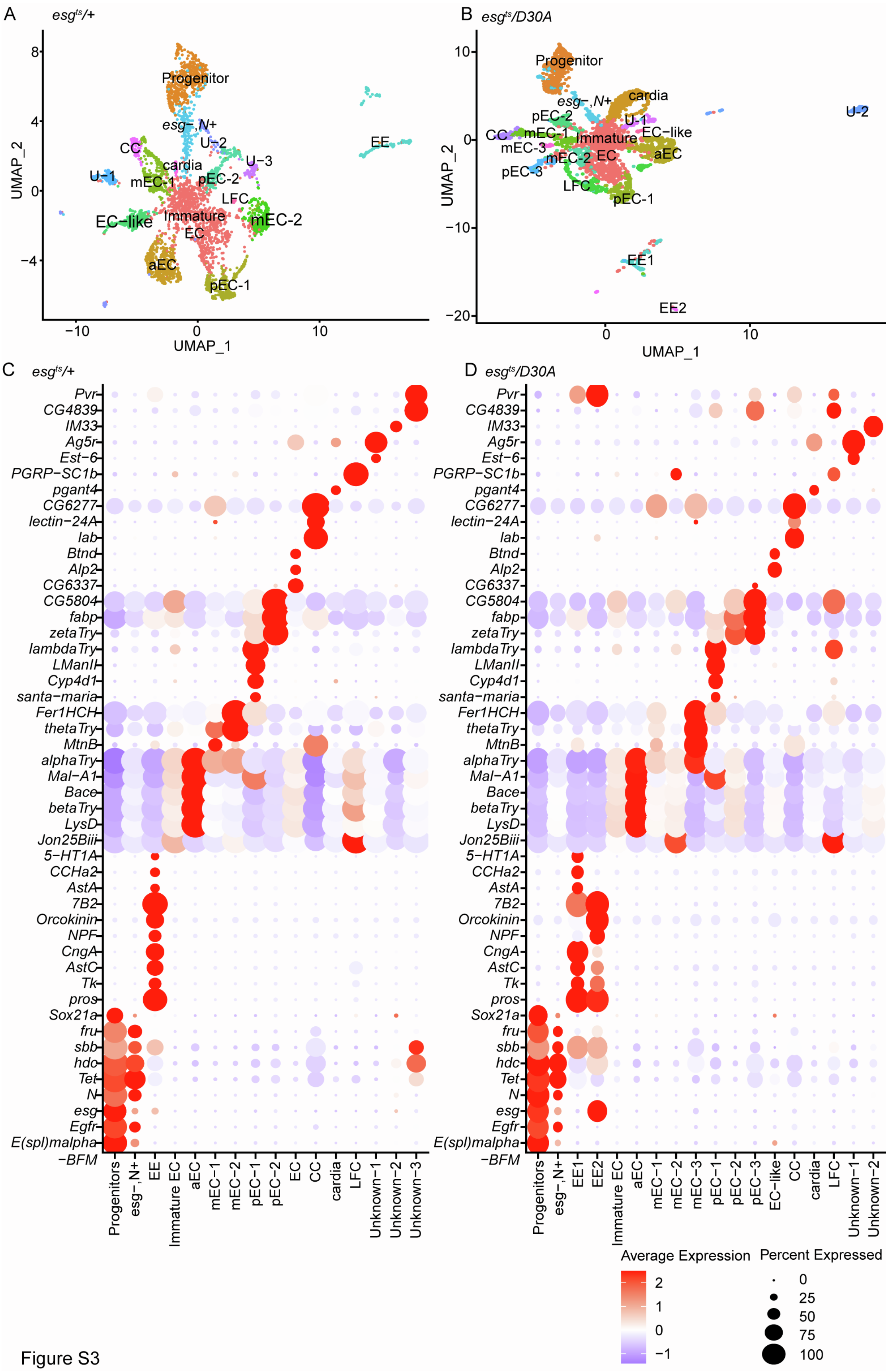
A
esg^ts/+
Progenitor
esg−,N+
U−2
CC
cardia
U−3
EE
U−1
mEC-1
pEC-2
LFC
Immature EC
mEC-2
EC−like
aEC
pEC-1
UMAP_1
UMAP_2
B
esg^ts/D30A
Progenitor
esg−,N+
cardia
pEC-2
U-1
U-2
CC
mEC-1
Immature
EC−like
mEC-3
aEC
mEC-2
EC
pEC-3
LFC
pEC-1
EE1
EE2
C
esg^ts/+
D
esg^ts/D30A
Pvr
CG4839
IM33
Ag5r
Est−6
PGRP−SC1b
pgant4
CG6277
lectin−24A
lab
Btnd
Alp2
CG6337
CG5804
fabp
zetaTry
lambdaTry
LManII
Cyp4d1
santa−maria
Fer1HCH
thetaTry
MtnB
alphaTry
Mal−A1
Bace
betaTry
LysD
Jon25Biii
5−HT1A
CCHa2
AstA
7B2
Orcokinin
NPF
CngA
AstC
Tk
pros
Sox21a
fru
sbb
hdc
Tet
N
esg
Egfr
E(spl)malpha−BFM
Progenitors
esg−,N+
EE
Immature EC
aEC
mEC−1
mEC−2
pEC−1
pEC−2
EC
CC
cardia
LFC
Unknown−1
Unknown−2
Unknown−3
EE1
EE2
mEC−3
pEC−3
EC−like
Average Expression
Percent Expressed

Figure S3

Figure S3. Transcriptional profile of progenitor-specific IMD inhibition on each epithelial cell type, Related to Figure 3.

A-B: Two dimensional UMAP projection of cell types isolated from female control esg^{ts}/+ intestines, and from female esg^{ts}/*D30A* intestines, color coded by cell type. U = unknown, CC = copper cells, EC = enterocyte, EE = enteroendocrine cell. **C-D:** Heatmap of IEC cluster markers colored by relative gene expression for flies of the indicated genotypes. The size of the dot indicates the proportion of cells in each cluster that expressed the indicated gene.

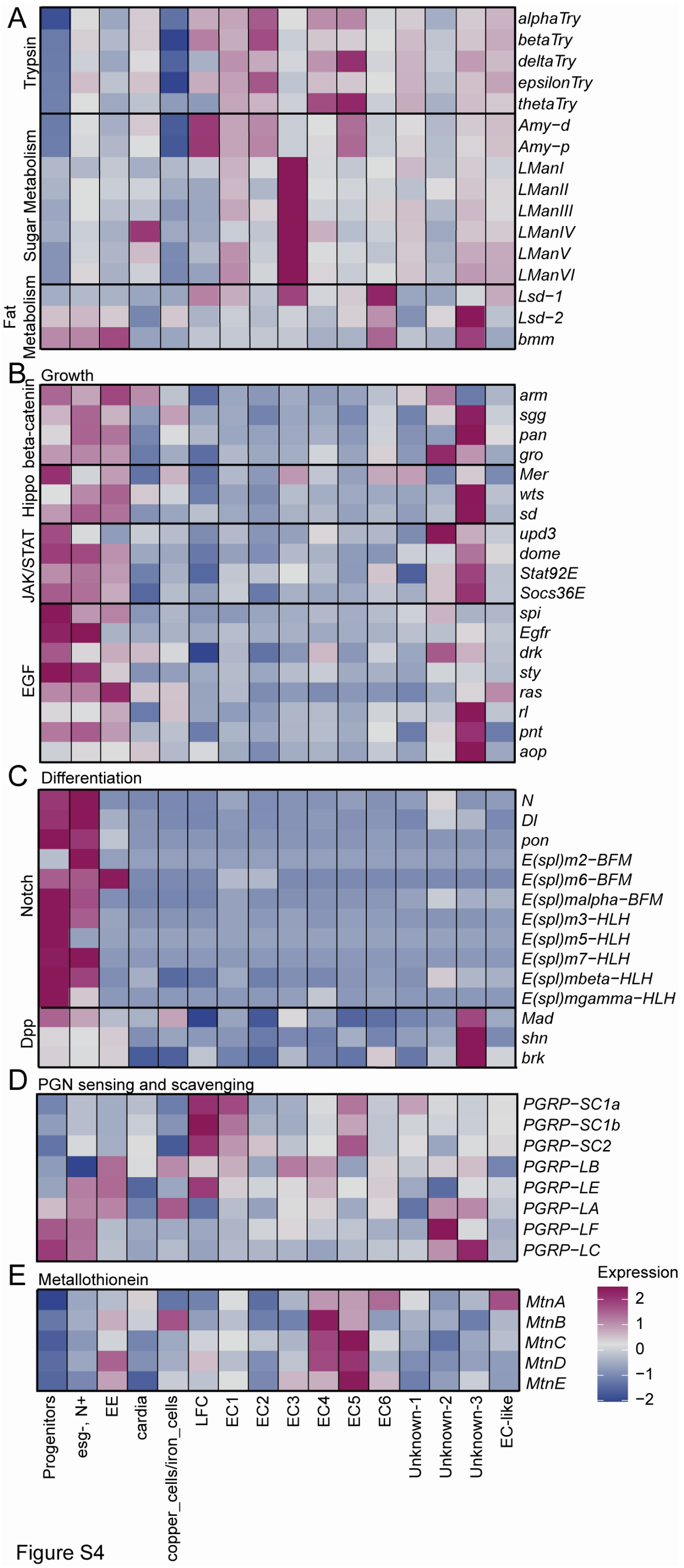
A
Trypsin
Sugar Metabolism
Fat Metabolism
alphaTry
betaTry
deltaTry
epsilonTry
thetaTry
Amy−d
Amy−p
LManI
LManII
LManIII
LManIV
LManV
LManVI
Lsd−1
Lsd−2
bmm
B
Growth
beta-catenin
Hippo
JAK/STAT
EGF
arm
sgg
pan
gro
Mer
wts
sd
upd3
dome
Stat92E
Socs36E
spi
Egfr
drk
sty
ras
rl
pnt
aop
C
Differentiation
Notch
Dpp
N
Dl
pon
E(spl)m2−BFM
E(spl)m6−BFM
E(spl)malpha−BFM
E(spl)m3−HLH
E(spl)m5−HLH
E(spl)m7−HLH
E(spl)mbeta−HLH
E(spl)mgamma−HLH
Mad
shn
brk
D
PGN sensing and scavenging
PGRP−SC1a
PGRP−SC1b
PGRP−SC2
PGRP−LB
PGRP−LE
PGRP−LA
PGRP−LF
PGRP−LC
E
Metallothionein
MtnA
MtnB
MtnC
MtnD
MtnE
Expression
2
1
0
−1
−2
Progenitors
esg-, N+
EE
cardia
copper_cells/iron_cells
LFC
EC1
EC2
EC3
EC4
EC5
EC6
Unknown-1
Unknown-2
Unknown-3
EC-like

Figure S4

Figure S4. Heatmap of metabolism, growth, Differentiation, Peptidoglycan detection, and oxidative stress responses regulators, Related to Figure 3.

A: Heatmap showing relative cluster-average expression of metabolic enzymes (Try = *Trypsin*, Amy = *Amylase*, LMan = Lsysomal alpha-mannosidase, Lsd = Lipid storage droplet, bmm = *brummer*) in each intestinal epithelial cell type of control *esg*^{ts}/+ flies. **B:** Heatmap showing relative cluster-average expression of prominent regulators of intestinal epithelial growth in each intestinal epithelial cell type of control *esg*^{ts}/+ flies. For this analysis, we focused on indicated components of the beta-catenin, Hippo, JAK/STAT, and Epidermal Growth Factor (EGF) pathways.

C: Heatmap showing relative cluster-average expression of prominent regulators of intestinal epithelial differentiation in each intestinal epithelial cell type of control *esg*^{ts}/+ flies. For this analysis, we focused on indicated components of the Notch and Decapentaplegic (Dpp, *Drosophila* ortholog of Bone Morphogenetic Protein) pathways. **D:** Heatmap showing relative cluster-average expression of prominent regulators of Peptidoglycan detection (*PGRP-LE, LC, LA, LF*), and peptidoglycan amidases (*PGRP-SC1a, SC1b, SC2, LB*) in each intestinal epithelial cell type of control *esg*^{ts}/+ flies. For this analysis, we focused on Peptidoglycan Recognition Proteins (PGRP) with detectable expression in the adult intestine. **E:** Heatmap showing relative cluster-average expression of prominent Metallothionein (*Mtn*) regulators of oxidative stress responses in each intestinal epithelial cell type of control *esg*^{ts}/+ flies.

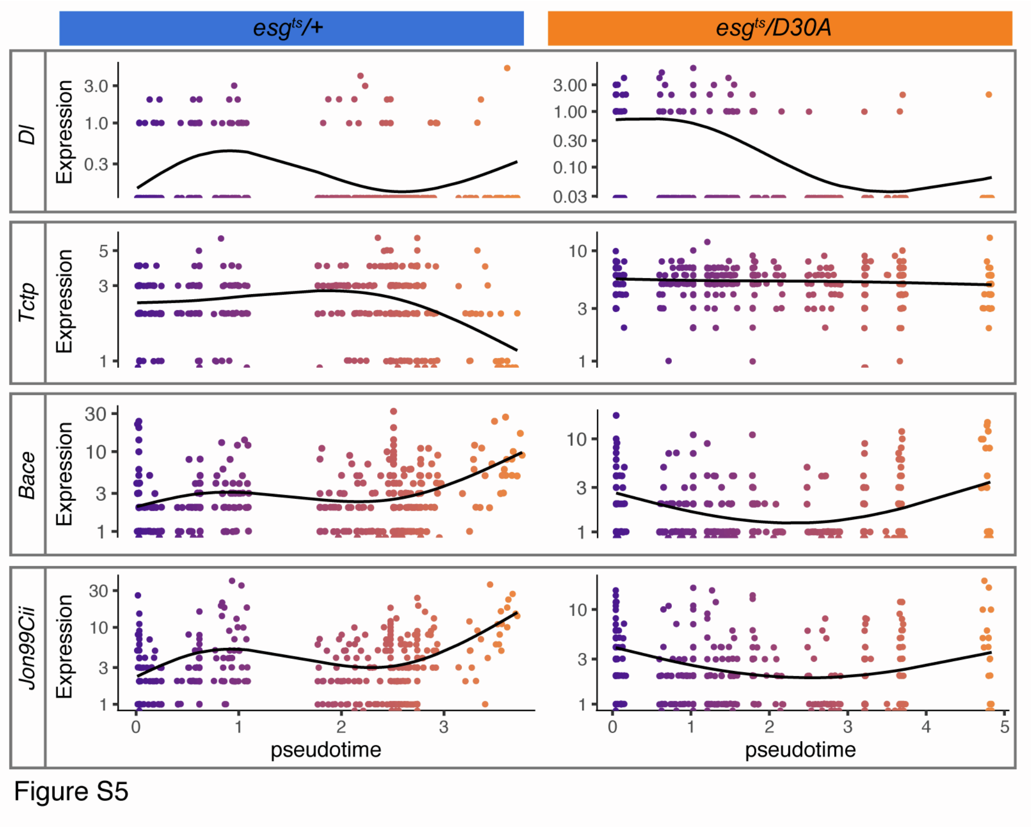

Figure S5. Inhibition of IMD disrupts progenitor cell expression trajectories, Related to Figure 4. Expression of the stem cell marker *Delta (Dl*), *Translationally-controlled tumor protein (Tctp),* and enterocyte markers *Bace* and *Jon99Cii* along pseudotime in *esg^{ts}*/+ and *esg^{ts}/D30A* progenitors. Dark purple marks cells at the beginning of pseudotime while orange marks cells late in pseudotime. Black lines show expression trend.

SUPPLEMENTAL TABLE

Table S1. Inactivation of IMD disrupted expression of 154 genes in progenitors, Related to Figure 1

	p_val	avg_logFC	pct.1	pct.2	p_val_adj	Cluster
**PGRPs**						
PGRP-SC2	0.007338224	0.34923964	0.85	0.827	1	aEC-1
PGRP-LA	0.050117136	-0.06789983	0.065	0.107	1	aEC-1
PGRP-LC	0.277498883	0.104399266	0.097	0.077	1	aEC-1
PGRP-SC1b	0.464238016	-0.06274809	0.053	0.066	1	aEC-1
PGRP-LB	0.162904492	-1.06574588	0.115	0.25	1	aEC-2
PGRP-SC2	0.181166722	0.612109524	0.793	0.75	1	aEC-2
PGRP-SC1b	0.40263009	0.75051183	0.081	0	1	aEC-2
PGRP-SC1a	0.546245626	0.427334596	0.23	0.125	1	aEC-2
PGRP-SC1a	0.00732842	0.26187825	0.222	0.121	1	mEC-1
PGRP-LE	0.01887176	-0.0697811	0.116	0.053	1	mEC-1
PGRP-SC1b	0.17777272	0.09942409	0.091	0.057	1	mEC-1
PGRP-SC2	0.95526117	-0.0728868	0.697	0.621	1	mEC-1
PGRP-SC1b	1.05E-10	0.87502663	0.55	0.156	1.31E-06	mEC-2
PGRP-SC1a	5.05E-10	1.06405117	0.667	0.266	6.33E-06	mEC-2
PGRP-LC	2.14E-05	0.0607342	0.167	0.027	0.26761866	mEC-2
PGRP-LB	2.52E-05	0.22971691	0.483	0.184	0.31613382	mEC-2
PGRP-LF	0.00038527	0.18178081	0.083	0.008	1	mEC-2
PGRP-SD	0.00038527	0.16098043	0.083	0.008	1	mEC-2
PGRP-SC2	0.0023101	0.52052858	0.933	0.98	1	mEC-2
PGRP-LA	0.008040939	-0.1992418	0.034	0.082	1	pEC-1
PGRP-SC1b	0.427890154	0.162931253	0.1	0.085	1	pEC-1
PGRP-LB	5.42E-08	0.67468029	0.36	0.109	0.000679152	pEC-2
PGRP-SC1b	0.452005775	0.069762129	0.08	0.06	1	pEC-2
PGRP-LE	0.581816348	-0.20016583	0.058	0.043	1	pEC-2
PGRP-SC1a	0.745670872	-0.27803406	0.182	0.174	1	pEC-2
PGRP-SC2	0.000989019	0.524798394	0.438	0.256	1	pEC-3
PGRP-LA	0.021084284	-0.39524465	0.177	0.286	1	pEC-3
PGRP-LE	0.106492034	0.173507048	0.285	0.203	1	pEC-3
PGRP-LF	0.169848264	-0.09222145	0.108	0.06	1	pEC-3
PGRP-LB	0.253406709	-0.14482782	0.515	0.391	1	pEC-3
PGRP-LC	0.316279848	-0.05088308	0.077	0.113	1	pEC-3
PGRP-LA	0.054696433	-0.18472686	0.312	0.43	1	pEC-4
PGRP-SC2	0.313823287	-0.10825062	0.729	0.767	1	pEC-4
PGRP-SC1a	0.332124379	0.051890641	0.094	0.058	1	pEC-4
PGRP-LB	0.342758952	-0.09222173	0.938	0.872	1	pEC-4
PGRP-LF	1.89E-07	0.448053148	0.093	0.016	0.002363045	EC-like-1
PGRP-SD	0.000297814	0.156637831	0.053	0.011	1	EC-like-1
PGRP-LB	0.004557101	0.067276621	0.233	0.129	1	EC-like-1
PGRP-LC	0.020479852	0.135834006	0.08	0.037	1	EC-like-1
PGRP-SC1a	0.13461254	-0.14660949	0.26	0.178	1	EC-like-1
PGRP-LF	0.021417831	0.106819364	0.056	0.004	1	EC-like-2
PGRP-LB	0.205049231	-0.71632539	0	0.083	1	EC-like-2
PGRP-SC1b	0.30118776	-0.57823276	0	0.057	1	EC-like-2
PGRP-LE	0.499728118	-0.19528606	0.056	0.026	1	EC-like-2
PGRP-SC2	0.77872758	-0.07259685	0.889	0.605	1	EC-like-2
**AMPs**						
Def	0.0214178	0.1057657	0.056	0.004	1	EC-like-2
Drsl3	0.031586	-0.1106446	0.117	0.043	1	mEC-2

Table S2. Expression of antimicrobial peptides, or peptidoglycan recognition proteins in differentiated enterocytes upon progenitor-specific IMD inhibition, Related to Figure 3.

EXPERIMENTAL PROCEDURES

Ecc15 Oral Infection

For oral infection with *Ecc15*, we incubated an overnight culture of *Ecc15* in LB (Difco^{TM} Luria Broth Base, Miller, 241420) supplemented with NaCl (4.75g Fisher Scientific, BP358-212 per 500mL of LB Broth base) at 29°C with shaking. Flies were starved (10 flies per vial) for 2 h before infection. *Ecc15* was pelleted at 1250g for 10 minutes at 4°C and supernatant decanted. The harvested bacterial pellet was re-suspended in residual LB and an equivalent volume of 5% sucrose in PBS. Flies were transferred into vials that contained a filter paper (Whatman^{TM}, Grade 3, 23mm, 1003-323) soaked with 150ml of the *Ecc15* culture on top of standard corn meal medium. Flies were infected for 16h at 29°C with 12h:12h light:dark cycle. To activate the GeneSwitch (GS) system we added 100µl RU486 (Mifepristone, M8046, Sigma) dissolved in 80% EtOH (5mg/ml) to the surface of standard fool and dried overnight prior to addition of flies. For controls, we added 100µl of 80% EtOH to the surface of standard fool and dried overnight prior to addition of flies. Flies were raised on treated food for 48h prior to infection.

Immunofluorescence

The number of PH-3 or Delta positive cells were analyzed with two-way ANOVA or unpaired Student's t-tests. We used previously described immunofluorescence protocols to visualize posterior midguts (56). In brief, we used anti-phospho-histone H3 (PH3, 1:1000, Millipore (Upstate), 06-570) immunofluorescence to quantify mitoses in the midguts, and anti-Delta (1:100; Developmental Studies Hybridoma Bank (DSHB) C594.9B) immunofluorescence to quantify stem cells in the R4/R5 region of the posterior midguts of virgin female flies that we raised at 29°C. We also used anti-prospero (1:100, DSHB), anti-armadillo (1:100, DSHB) as primary antibodies and Hoechst 33258 (1:500; Molecular Probes) for DNA staining. Secondary antibodies used: goat anti-mouse Alexa Fluor 568 (1:500; Invitrogen), goat anti-rabbit 488 (1:500; Invitrogen). Tissue was mounted in Fluoromount (Sigma-Aldrich F4680) and posterior midguts were visualized with a spinning disk confocal microscope (Quorum WaveFX;

Quorum Technologies Inc.). Images were collected as Z-slices and processed with Fiji software to generate a Z-stacked image.

Isolation of progenitor cell and RNA extraction

Progenitor cells were isolated by fluorescence activated cell sorting (FACS) as previously described by Dutta et al. (2013). Flies were raised at 29°C for 10 days. 100 fly guts per sample were dissected (malphighian tubules, foreguts, hindguts and crops removed) and placed into ice-cold 1XPBS/DEPC-treated water. Guts were dissociated with 1mg/ml of elastase at 27°C with periodic pipetting for 1h. GFP-positive progenitor cells were collected based on GFP fluorescence and size with a BD FACSAriaIII sorter. Cells were pelleted at 1200g for 5 minutes at 4°C and then resuspended in 500µl Trizol. Samples were stored at -80°C until all three biological replicates were collected. RNA was isolated via a standard Trizol-chloroform extraction and the RNA was sent on dry ice to the Lunenfeld-Tanenbaum Research Institute (Toronto, Canada) for library construction and sequencing. The sample quality was evaluated using Agilent Bioanalyzer 2100. TaKaRa SMART-Seq v4 Ultra Low Input RNA Kit for Sequencing was used to prepare full length cDNA. The quality and quantity of the purified cDNA was measure with Bioanalyzer and Qubit 2.0. Libraries were sequenced on the Illumina HiSeq3000 platform.

Preparation of Single Cell Suspension for single cell RNAseq

Single-cell suspension preparation method were followed (Hung et al., 2018) with a few modifications. Flies were raised for 10 days at 29°C. Five guts were dissected at one time and moved to 1% BSA in PBS/DEPC-treated water. Once twenty-seven guts were dissected, we transferred the guts to 200ml 1XPBS/DEPC-treated water on the back side of a glass dissection plate (PYREX, 7220-85) and chopped with scissors. After mechanically fragmenting the tissue, it was transferred to a 1.5 ml tube containing 100ml 1XPBS/DEPC-treated water then enzymatically digested with elastase (final concentration 1mg/ml) at 27°C for 40 min with gentle pipetting every 10 min. The

single cell suspension was pelleted at 300g for 15 min at 4°C and cell pellet resuspended in 200ml 0.04%BSA in 1XPBS/DEPC-treated water. The cell suspension was filtered through a 70μm filter (300g for 1 min at 4°C).

Live cells were collected using OptiPrepTM Density Gradient Medium (SIGMA, D1556-250ML) using the OptiPrepTM Application Sheet C13 protocol. Briefly, a 40% (w/v) iodixanol working solution was prepared with 2 volumes of OptiPrepTM and 1 volume of 0.04 %BSA in 1XPBS/DEPC-treated water. This working solution was used to prepare a 22% (w/v) iodixanol solution in the same buffer. One volume of working solution was carefully mixed with 0.45 volume of cell suspension by gently inversion. The cell suspension/working solution mixture was transferred to a 15ml conical tube then topped up to 6 ml with working solution. The working solution/cell suspension was overlaid with 3 ml of the 22% (w/v) iodixanol and the 22% iodixanol layer was overlaid with 0.5 ml of 0.04 %BSA in 1XPBS/DEPC. Viable cells were separated by density gradient created by centrifuging at 800 g for 20 min at 20°C. Viable cells were harvested from the top interface (~500ul) and then diluted in 2 volumes (1ml) of 0.04 %BSA in 1XPBS/DEPC-treated water. The iodixanol was removed by pelleting live cell suspension at 300 g for 10 min at 4°C. Supernatant was decanted and cells were resuspended in the leftover 0.04 %BSA in 1XPBS/DEPC-treated water. Viability and concentration were measured by 0.4% trypan blue (Gibco, 15250-061) and hemocytometer. Libraries were generated with a 10X Genomics Single-cell Transcriptome Library kit.

Bioinformatics

For purified progenitor RNAseq studies, we obtained approximately 6 million reads per biological replicate. We used FASTQC to evaluate the quality of raw, paired- end reads, and trimmed adaptors and reads of less than 36 base pairs in length from the raw reads using Trimmomatic version 0.36. We used HISAT2 version 2.1.0 to align reads to the *Drosophila* transcriptome- bdgp6, and converted the resulting BAM files to SAM flies using Samtools version 1.8. We counted converted files using Rsubread version 1.24.2 and loaded them into EdgeR. In EdgeR, we filtered genes with counts less than 1 count per million and normalized libraries for size. Normalized libraries were used to call genes that were differentially expressed among treatments. Genes with P-value < 0.05 and FDR < 0.05

were defined as differentially expressed. Principle component analysis was performed on normalized libraries using Factoextra version 1.0.5, and Gene Ontology enRIchment anaLysis and visuaLizAtion tool (GORILLA) was used to determine Gene Ontology (GO) term enrichment. Specifically, differentially expressed genes were compared in a two-list unraked comparison to all genes output from edgeR as a background set, and redundant GO terms were removed.

For single cell analysis, Cell Ranger v3.0 was used to align sequencing reads to the *Drosophila* reference transcriptome (FlyBase, r6.30) and generate feature-barcode matrices. These matrices were analyzed using the Seurat R package (version 3.2.3). Cells possessing <500 UMIs or >2500 UMIs were removed to reduce the number of low- quality cells and doublets. Seurat was then used to normalize expression values and perform integrated data cell clustering at a resolution of 0.5 with 15 principal components. Clusters were identified based on known markers and previous single-cell analysis of the *Drosophila* intestine (https://www.flyrnai.org/scRNA/). For GO term analysis of single cell data, Seurat was used to integrate esg^{ts}/+ and esg^{ts}/D30A datasets and generate lists of differentially expressed genes for each cluster. Both up- and down-regulated gene lists (p-value cut-off <0.05) were analyzed in GOrilla to determine GO term enrichment. Differentially expressed genes were compared in a two-list unranked comparison to all genes identified in the single- cell dataset. GO terms were then analyzed in REVIGO (REduce and VIsualize Gene Ontology) to remove redundant GO terms. Top enriched GO terms are shown for each cluster, as well as those same GO terms found in other clusters. EE subset analysis was followed at Guo et al. (2019).

For Pseudotime analysis we used Monocle3 (version 0.2.0). Specifically, we converted the existing Seurat data from each genotype separately into a Monocle cell data set of midgut epithelial cells and performed trajectory analysis. We manually assigned the root node of the trajectory to the node at the tip of the Progenitor cluster for each genotype. We then subset the trajectory branch that explains pseudotime within the Progenitor population to perform all subsequent gene level analysis. Here, we manually assessed expression of genes along pseudotime

with known functions in ISC identity, division, and differentiation including genes that were differentially expressed based off our Seurat analysis.

Data availability

Gene expression data have been submitted to the NCBI GEO database (GEO: SuperSeries GSE141897 (GSE171001 and GSE141896)). Single cell gene expression data for *esg*^{ts}*/+* flies (https://singlecell.broadinstitute.org/single_cell/study/SCP1696/single-cell-expression-data-for-d-melanogaster-wild-type-intestines) and for *esg*^{ts}*/D30A* flies (https://singlecell.broadinstitute.org/single_cell/study/SCP1699/single-cell-expression-data-for-d-melanogaster-intestines-with-immune-deficient-progenitor-cells#study-summary) are available for visualization on the Broad Institute Single Cell Portal.
